# Genome, transcriptome and secretome analyses of the antagonistic, yeast-like fungus *Aureobasidium pullulans* to identify potential biocontrol genes

**DOI:** 10.15698/mic2021.08.757

**Published:** 2021-06-08

**Authors:** Maria Paula Rueda-Mejia, Lukas Nägeli, Stefanie Lutz, Richard D. Hayes, Adithi R. Varadarajan, Igor V. Grigoriev, Christian H. Ahrens, Florian M. Freimoser

**Affiliations:** 1 Agroscope, Research Division Plant Protection, Müller-Thurgau-Strasse 29, 8820 Wädenswil, Switzerland.; 2 Agroscope, Competence Division Method Development and Analytics, Müller-Thurgau-Strasse 29, 8820, Wädenswil, Switzerland.; 3 U.S. Department of Energy Joint Genome Institute (JGI), Lawrence Berkeley National Laboratory, 1 Cyclotron Rd., Berkeley, California 94720, USA.; 4 Department of Plant and Microbial Biology, University of California Berkeley, Koshland Hall, Berkeley, CA, USA.; 5 SIB, Swiss Institute of Bioinformatics, Wädenswil, Switzerland.

**Keywords:** antagonism, Aureobasidium, biocontrol, Fusarium, genome, proteome, secretome, transcriptome, yeast

## Abstract

*Aureobasidium pullulans* is an extremotolerant, cosmopolitan yeast-like fungus that successfully colonises vastly different ecological niches. The species is widely used in biotechnology and successfully applied as a commercial biocontrol agent against postharvest diseases and fireblight. However, the exact mechanisms that are responsible for its antagonistic activity against diverse plant pathogens are not known at the molecular level. Thus, it is difficult to optimise and improve the biocontrol applications of this species. As a foundation for elucidating biocontrol mechanisms, we have *de novo* assembled a high-quality reference genome of a strongly antagonistic *A. pullulans* strain, performed dual RNA-seq experiments, and analysed proteins secreted during the interaction with the plant pathogen *Fusarium oxysporum*. Based on the genome annotation, potential biocontrol genes were predicted to encode secreted hydrolases or to be part of secondary metabolite clusters (e.g., NRPS-like, NRPS, T1PKS, terpene, and β-lactone clusters). Transcriptome and secretome analyses defined a subset of 79 *A. pullulans* genes (among the 10,925 annotated genes) that were transcriptionally upregulated or exclusively detected at the protein level during the competition with *F. oxysporum*. These potential biocontrol genes comprised predicted secreted hydrolases such as glycosylases, esterases, and proteases, as well as genes encoding enzymes, which are predicted to be involved in the synthesis of secondary metabolites. This study highlights the value of a sequential approach starting with genome mining and consecutive transcriptome and secretome analyses in order to identify a limited number of potential target genes for detailed, functional analyses.

## INTRODUCTION

*Aureobasidium pullulans* belongs to the Dothideales, not the classical yeast order Saccharomycetales, and exhibits both yeast-like and hyphal growth morphology [1]. Nevertheless, the species is often referred to as a yeast, black yeast, or yeast-like fungus and biocontrol species. Fungi of the genus *Aureobasidium*, and in particular *A. pullulans*, are frequently isolated from phyllosphere and soil samples worldwide. Their phenotypic plasticity and tolerance of harsh environmental conditions are likely the reason for the competitiveness of these ascomycetes in a wide range of ecological niches [1, 2]. The genomes of 50 *A. pullulans* strains from different environments and geographical regions revealed a homogenous population structure and limited genome variability. It has thus been concluded that *A. pullulans* is an efficiently spreading and recombining fungus [3].

Different strains of *A. pullulans* produce a wide array of well-characterized metabolites (e.g., pullulan, lanthipeptides, liamocin, aureobasidin), extracellular enzymes, siderophores and toxins [4–10]. Selected enzymes with proteinase, cellulase, lipase, glucanase, mannanase and chitinase activities, among others, have been characterized [9, 11] (and references therein). The diverse collection of metabolites and enzymatic functions could be an explanation for the ecological versatility and antagonistic activity of *A. pullulans*. The metabolites and enzymatic activities are also the underlying reason for significant biotechnological interest. *Aureobasidium* species are studied, for example, as sources of proteins for food supplements and enzymes and used for the production of pullulan [4, 12], a polysaccharide used as a food additive. The applications in agriculture and biotechnology also provoked and justified substantial interest in more fundamental molecular studies. Transformation protocols and CRISPR/Cas 9 approaches have been established for some strains [13]. Genome sequences of different *Aureobasidium* species have also been analysed and are available as a valuable basis for functional and applied studies. The genomes of four *Aureobasidium* species, *A. melanogenum, A. namibiae, A. pullulans*, and *A. subglaciale*, were analysed by the same pipeline used here, revealing interesting features, including numerous secondary metabolite biosynthesis clusters (i.e., 37 for *A. melanogenum* and *A. subglaciale*), an unexpectedly large number of aquaporin-like genes (11-12 genes in each species), and an enrichment of sugar transporters [14].

The species *A. pullulans* is also known as an effective biocontrol agent that is thought to employ a range of mechanisms to suppress the growth of other microorganisms [9]. These mechanisms include direct competition [15–17], the secretion of enzymes and secondary metabolites [4–8, 18, 19], the production of volatile compounds [20–25], as well as the induction of resistance in crop plants [26, 27]. Many *Aureobasidium* isolates show strong biocontrol activity against destructive plant diseases such as moulds, rots and blights [28–35]. The biocontrol products BlossomProtect^™^ and Botector^®^ are based on two *A. pullulans* strains and registered to control fireblight and grey mould, respectively. However, with respect to biocontrol mechanisms, *A. pullulans* is surprisingly under-studied at the molecular level [9]. An alkaline serine protease from the *A. pullulans* strain PL5 was identified and biochemically characterised [18, 19]. The purified enzyme reduced the conidia germination rate and the germ tube length of different plant pathogens such as *Alternaria alternata, Botrytis cinerea, Penicillium expansum, Monilinia fructicola* and *Monilinia laxa*. However, the corresponding gene has not been deleted or overexpressed to further study its contribution to biocontrol activity. An alkaline protease was also identified in the marine *A. pullulans* strain HN2-3 [36]. Expressing its encoding gene in *Yarrowia lipolytica* conferred protease activity and the ability to generate bioactive peptides from different protein sources, but biocontrol activity has not been studied.

In the work presented here, we studied the *A. pullulans* isolate NBB 7.2.1, which was one of the strongest biocontrol organisms in an *in vitro* screen [37]. As a basis for functional studies, we *de novo* assembled a high-quality reference genome using long and short sequencing reads and used comparative genomics to evaluate the metabolic potential of this soil isolate of *A. pullulans*. We employed transcriptome and proteome analyses to identify potential biocontrol factors in confrontation assays with *Fusarium oxysporum*. The analyses focused on the putative secreted proteome, degrading enzymes, and on the production of secondary metabolites. Noteworthy was a large number of genes and gene clusters encoding secreted enzymes, biosynthetic proteins of antimicrobial compounds and peptides. Many genes were significantly upregulated by *A. pullulans* in the presence of *F. oxysporum.* A differential analysis of the secreted proteome also identified a subset of proteins that was upregulated or only detected during the interaction with *F. oxysporum*. This comprehensive study of the *A. pullulans* NBB 7.2.1 genome and the subsequent transcriptome and secretome analyses thus provided a subset of potential biocontrol genes. Detailed studies at the molecular level will determine the contribution of these genes to the biocontrol phenotype of *A. pullulans* NBB 7.2.1 and antagonistic yeasts in general.

## RESULTS

**Figure 1:**
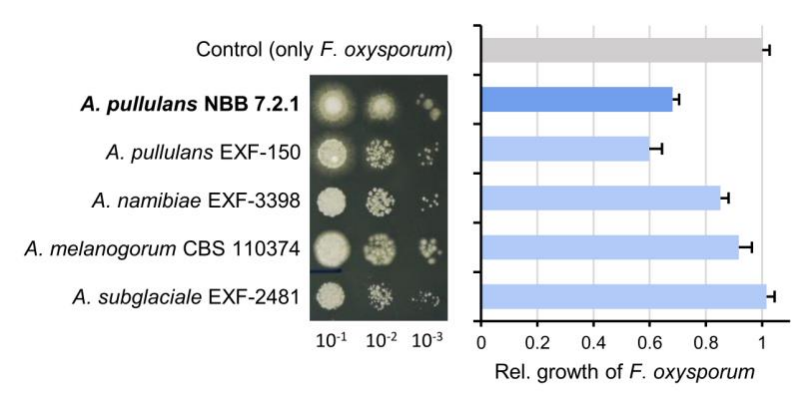
FIGURE 1: Comparison of five *Aureobasidium* species with respect to growth on agar plates and antagonistic activity. The different *Aureobasidium* species grow at comparable rates in spot assays, but show differences in antagonistic activity against the plant pathogenic fungus *F. oxysporum* NRRL 26381/CL57. Overnight cultures of the five strains were diluted to OD_600_=0.1 and three serial 1:10 dilutions were spotted on PDB agar (left). Competition assays were performed with highly diluted *Aureobasidium* samples (OD_600_ = 0.001) and the area of *F. oxysporum* growth was measured to reveal the differences in their antagonistic activity. The relative growth of *F. oxysporum* was reduced in competition with both *A. pullulans* strains (NBB 7.2.1 and EXF-150), while the other *Aureobasidium* species were less antagonistic.

The species *A. pullulans* and the *A. pullulans* strain NBB 7.2.1 are strongly antagonistic against several plant pathogens and highly competitive against other yeasts on apple fruits [9, 37, 38]. Here, *A. pullulans* NBB 7.2.1 exhibited comparable growth and antagonistic activity as *A. pullulans* EXF-150, but inhibited *F. oxysporum* NRRL 26381/CL57 more strongly than *A. melanogenum* CBS 110374, *A. namibiae* CBS 147.97, and *A. subglaciale* EXF-2481 (**Figure 1**). These differences were revealed when using diluted *Aureobasidium* inocula (i.e., with low colony forming units). As a foundation for mechanistic studies and to elucidate biocontrol factors, we first created a high-quality reference genome and compared it to previously sequenced *Aureobasidium* species that are not widely described as biocontrol organisms. Subsequently, we performed transcriptome analyses and identified secreted proteins allowing to further prioritise the potential biocontrol genes.

### Genome analysis of *A. pullulans* NBB 7.2.1

We sequenced the genome of *A. pullulans* NBB 7.2.1 using PacBio Sequel long read (mean read length 10,126 bp, 35x-207x coverage, 98.61% of reads mapped to final assembly) and Illumina short read sequencing technologies (2x300 bp, 29x coverage, 100% of reads mapped to final assembly) (Supplementary Table 1). The final *A. pullulans* NBB 7.2.1 genome assembly had a size of 28.41 Mbp and consisted of twelve contigs, presumably chromosomes, and a circular, complete mitogenome (Supplementary Table 2). Annotation of this contiguous, high quality genome by JGI predicted 10,925 genes (with an average gene and protein length of 1,569 bp and 479 aa, respectively; on average 2.6 introns/gene; https://mycocosm.jgi.doe.gov/AurpulNBB1/AurpulNBB1.info.html) (Supplementary Table 3).

#### Genome analysis and pan-genome clustering

In order to gain a better understanding of the peculiarities of the *A. pullulans* NBB 7.2.1 genome, a comparative analysis with four other *Aureobasidium* genomes that were previously annotated by the same pipeline [14] was performed. The genomes of *A. melanogenum* CBS 110374, *A. namibiae* CBS 147.97), *A. pullulans* EXF-150, and *A. subglaciale* EXF-2481 are of comparable size (26.20 Mbp, 25.43 Mbp, 29.62 Mbp, and 25.80 Mbp, respectively) and contain similar numbers of predicted genes (10,594, 10,266, 11,866, and 10,809, respectively) as *A. pullulans* NBB 7.2.1 (28.41 Mbp; 10,925 genes; https://mycocosm.jgi.doe.gov/aureobasidium). All five genome annotations comprised a comparable number of KEGG terms (Supplementary Table 3).

**Figure 2:**
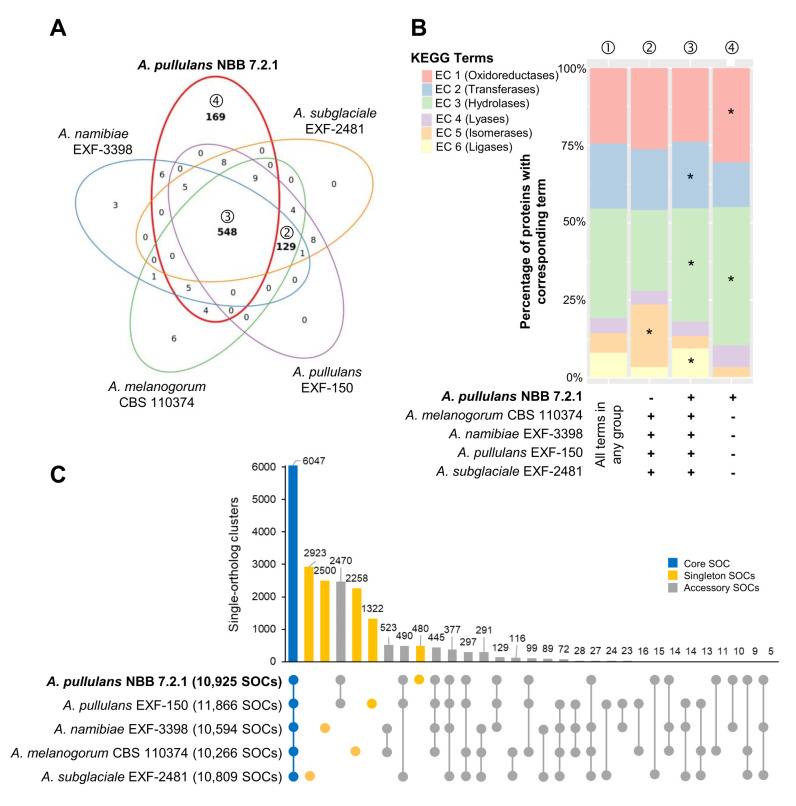
FIGURE 2: Whole genome comparison of five *Aureobasidium* species. Gene annotations reveal that the *A. pullulans* NBB 7.2.1 genome contains more unique KEGG terms than the genomes of four other *Aureobasidium* strains. **(A)** KEGG term distribution among annotated genes of five *Aureobasidium* species. 548 terms were commonly found in all species, but a substantial number of terms (169) was unique for the *A. pullulans* NBB 7.2.1 genome or specifically lacking in this genome (129 terms; all bold). **(B)** The relative number of genes annotated to one of the six main enzyme classes (EC 1-6; oxidoreductases, transferases, hydrolases, lyases, isomerases, ligases) for all KEGG terms in the five *Aureobasidium* species and the subsets described in A. The enzyme classes highlighted with an asterisk (*) are significantly overrepresented (adj. p-value≤0.05) in the respective group (as compared to their frequency among all KEGG terms found in any of the five *Aureobasidium* genomes (①)). Results are shown for those genes with terms shared among all genomes except *A. pullulans* NBB 7.2.1 (②), shared among all five genomes (③), or only present in *A. pullulans* NBB 7.2.1 (④). **(C)** Pan-genome clustering of five *Aureobasidium* genomes visualized using an Up Set bar diagram [96]. The *A. pullulans* NBB 7.2.1 genome contained fewer (480) unique gene models than the four other *Aureobasidium* genomes.

Most of the KEGG terms associated with the five *Aureobasidium* genomes were shared (548 terms), but a high number (169 terms) was either uniquely found for genes in the *A. pullulans* NBB 7.2.1 genome or specifically lacking for genes in this genome (129 terms; **Figure 2A**). Similar analyses for GO, IPR or KOG terms showed comparable results (Supplementary Figure S1). Among the genes with KEGG terms only lacking in *A. pullulans* NBB 7.2.1 (129 terms), isomerases (EC 5) were significantly enriched (adj. p-value < 0.001) as compared to their frequency among all KEGG terms identified for the five genomes. Among the terms only present in the *A. pullulans* NBB 7.2.1 genome (169 terms), putative hydrolases (EC 3; adj. p-value < 0.001) and oxidoreductases (EC 1; adj. p-value 0.012) were significantly enriched. Hydrolases (EC 3; adj. p-value < 0.001), in addition to transferases (EC 2; adj. p-value 0.012) and ligases (EC 6; adj. p-value < 0.001), were enriched among the terms shared in all five genomes (548 terms; **Figure 2B**).

The Pangloss pan-genome was established using the published workflow and reflects a single-ortholog clustering (SOC), where each cluster contains at most one gene from each species [39, 40]. Comparison to Markov gene families highlights which SOCs are related by multi-copy paralogy, or might be similar to other SOCs, and which pairwise SOCs and singletons truly contain unique sequence. 6,047 SOCs were shared by all five *Aureobasidium* genomes (out of 10,266-11,866 gene models for the five strains), whereas 15,090 accessory SOCs were only present in some of the five strains (5,603 partial clusters present in two to four strains and 9,483 gene models unique to one of the five strains; **Figure 2C**). The *A. pullulans* NBB 7.2.1 genome contained only 480 unique SOCs, as compared to 2,923, 2,500, 2,258, and 1,322 SOCs for *A. subglaciale* EXF-2481, *A. melanogenum* CBS 110374, *A. namibiae* CBS 147.97, and *A. pullulans* EXF-150, respectively. For *A. pullulans* NBB 7.2.1, the subset of true singleton SOCs (those without paralogs) was 116, most of which did not have a functional annotation. The strongest pairwise clustering was observed between the *A. pullulans* NBB 7.2.1 and *A. pullulans* EXF-150 genomes, which had 2,470 SOCs in common (by far the closest pairwise similarity among any of the five genomes). Of these, 535 SOCs represented pairs of single-copy orthologs, while an additional 55 were found in multi-copy gene families with only *A. pullulans* NBB 7.2.1 and *A. pullulans* EXF-150 genes.

**Figure 3:**
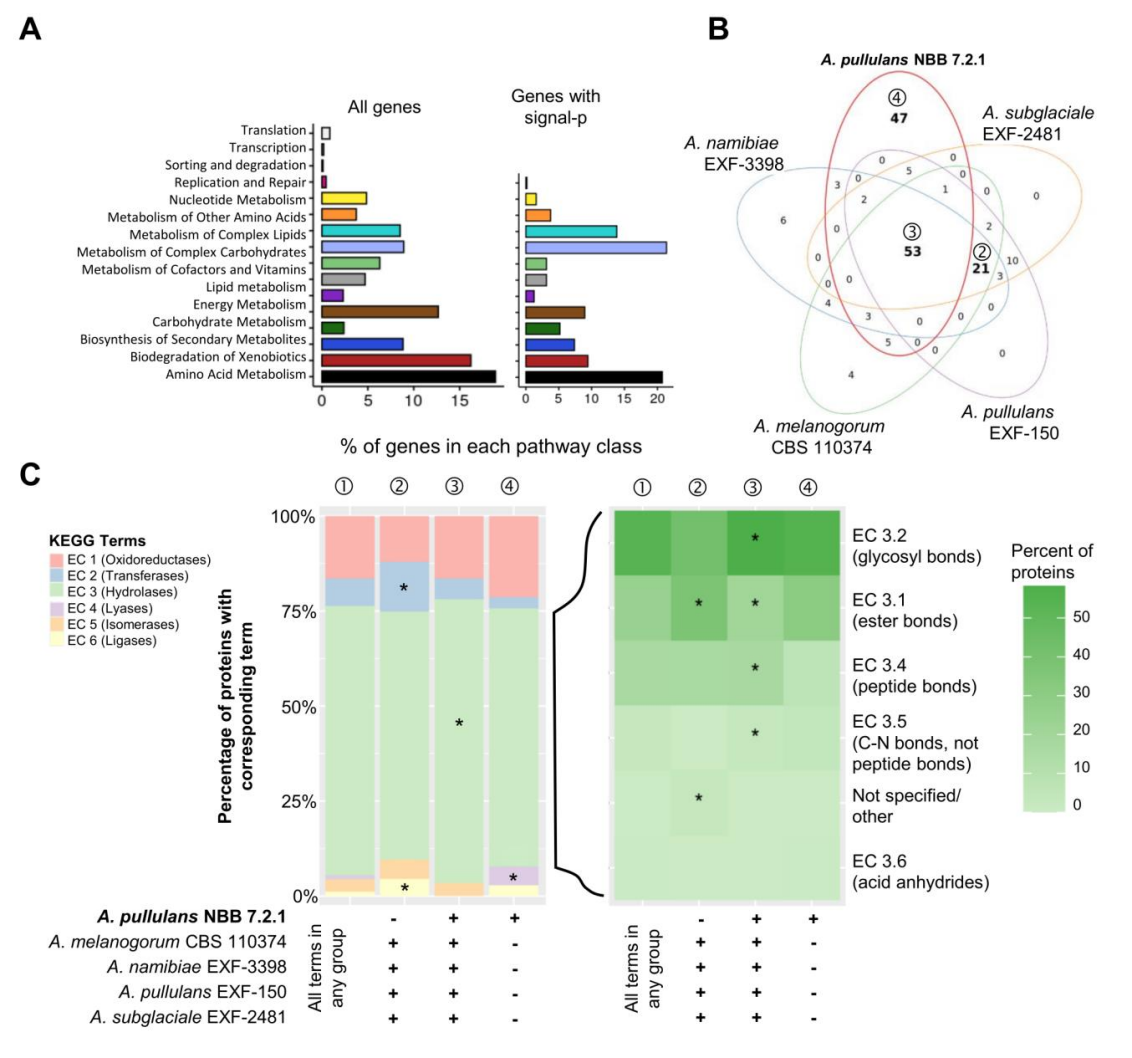
FIGURE 3: Genome mining of *Aureobasidium* genomes to identify potential biocontrol genes. Functional annotations of the five *Aureobasidium* genomes (obtained from the DOE-JGI MycoCosm) identified a plethora of genes and gene clusters that may contribute to biocontrol activity. **(A)** Relative percentage of *A. pullulans* NBB 7.2.1 genes (for the 4019 genes with a KEGG annotation or only the subset containing a predicted signal peptide) assigned to the different KEGG pathways. **(B)** KEGG term distribution among the five *Aureobasidium* genomes for all annotated genes containing a predicted signal peptide. 53 terms were commonly found in all species (③), while 47 terms were unique for the *A. pullulans* NBB 7.2.1 genome (④) and 21 were exclusively found in the other genomes (②) (bold numbers in Venn diagram). **(C)** The relative percentage of genes with a predicted signal peptide annotated to belong to one of the six main enzyme classes (EC 1-6; oxidoreductases, transferases, hydrolases, lyases, isomerases, ligases). Results are shown for genes with terms that were found in any of the five genomes (①) and for those shared in all genomes except *A. pullulans* NBB 7.2.1 (②), shared among all five genomes (③), or only present in *A. pullulans* NBB 7.2.1 (④). The enzyme classes highlighted with an asterisk were significantly overrepresented (adj. p-value≤0.05) in the respective group. Among the predicted hydrolase genes with a signal peptide, esterases (EC 3.1), glycosylases (EC 3.2), and peptidases (EC 3.4) were by far the most frequent and significantly overrepresented in genes with terms shared by all five *Aureobasidium* genomes.

Overall, almost 50% of the *A. pullulans* NBB 7.2.1 genes with KEGG terms were annotated to function in the metabolism of amino acids, carbohydrates, complex carbohydrates, and complex lipids (**Figure 3A**). These broad categories were the most abundant in the genome overall, but also among the subset of genes with a predicted signal peptide. Many of these potentially secreted gene products encoded hydrolases. In all five *Aureobasidium* genomes compared here, they were significantly overrepresented in contrast to hydrolases without a predicted signal peptide. As secreted hydrolases have also been implicated in biocontrol activity, they are discussed in more detail below.

#### Genes involved in secondary metabolite production

Secondary metabolites are compounds with low molecular weight that are not essential for growth, development and reproduction. They have diverse chemical compositions and biological functions. Many of them exhibit a role in fungal antagonism [8]. We used KEGG annotations and antiSMASH predictions, which rely on different principles to identify genes involved in secondary metabolite production. The KEGG annotation of the *A. pullulans* NBB 7.2.1 genome predicted 85 genes (2.1% of all genes with a KEGG annotation) to encode proteins involved in the synthesis of secondary metabolites. Of these 85 genes, 33 were predicted to contain a secretion signal (**Figure 3A**). Additionally, 25 distinct secondary metabolite clusters, comprising 27 biosynthetic genes, were found using the fungal antiSMASH v.5.1.2 online tool [41, 42] (Supplementary Tables 4 and 5). Only three core biosynthetic genes were also predicted by the KEGG annotation. The other four *Aureobasidium* genomes (*A. melanogenum* CBS 110374, *A. namibiae* CBS 147.97, *A. pullulans* EXF-150, and *A. subglaciale* EXF-2481) contained 19, 18, 25, and 25 such clusters, respectively. We noted that some of the biosynthesis enzymes identified in this search had no predicted function in the original annotation. The antiSMASH analysis predicted ten non-ribosomal peptide-like synthase clusters (NRPS-like), five polyketide synthase (PKS) clusters, four terpene clusters, three non-ribosomal peptide synthase clusters (NRPS) and one β-lactone cluster (Supplementary Table 5). Additionally, two clusters containing more than one core biosynthetic gene were detected: one with a NRPS and a polyketide synthase and one with a NRPS and a β-lactone synthase. Out of the products from all clusters identified, two had similarity with described biosynthetic enzymes: the polyketide synthase of the *A. alternata* ACRL toxin cluster, implicated in host-selective pathogenicity, showed 58% amino acid identity with a type 1 PKS in our genome (Protein ID 78529). This cluster is also present in *A. pullulans* (EXF-150) and in *A. subglaciale* (EXF-2481) [43]. Similarly, a melanin synthase from *Bipolaris oryzae* showed 67% amino acid identity with a different type 1 PKS (Protein ID 58443) [44] present in all five strains. The biosynthetic products of the other clusters could not be predicted based on the sequence comparisons.

#### Genes with a predicted signal peptide

Proteins with a predicted signal peptide likely represent secreted proteins. These are of particular interest, because they may directly mediate antagonistic activity. Among the 10,925 predicted *A. pullulans* NBB 7.2.1 gene models, 1,044 had a predicted signal peptide and may thus encode secreted proteins (SigP probability > 0.95 according to Mycocosm annotation; Supplementary Table 6). Across the five *Aureobasidium* genomes, and for the genes comprising a predicted signal peptide, 53 KEGG terms were common to all five genomes, while 47 and 21 were only present in *A. pullulans* NBB 7.2.1 or specifically lacking in this strain's genome, respectively (**Figure 3B**). The majority (248) of the 330 proteins with a predicted signal peptide and a KEGG term in *A. pullulans* NBB 7.2.1 were annotated as hydrolases (EC 3; >70% of all genes). Oxidoreductase (EC 1), transferase (EC 2), lyase (EC 4), isomerase (EC 5), and ligase (EC 6) genes were less frequent. Interestingly, transferase and ligase genes were significantly overrepresented among terms that were lacking in the *A. pullulans* NBB 7.2.1 genome (adj. p-values 0.006 and 0.002, respectively), while lyase genes were overrepresented among the 47 genes with unique KEGG terms in the *A. pullulans* NBB 7.2.1 genome (adj. p-value 0.014; **Figure 3C**). The large group of the predicted hydrolase genes with a signal peptide were significantly enriched among those shared by all five genomes (adj. p-value < 0.001). Enzymes predicted to act on ester (EC 3.1; adj. p-value < 0.001), N-glycosidic (EC 3.2; adj. p-value < 0.001), and peptide bonds (EC 3.4; adj. p-value < 0.001) were by far the most frequent and also significantly overrepresented in genes with terms shared by all genomes (**Figure 3C**). None of the hydrolase subclasses was uniquely enriched in genes specific for the *A. pullulans* NBB 7.2.1 genome, but those hydrolases predicted to act on ester bonds (EC 3.1 adj. p-value < 0.001) and unspecified hydrolases were significantly enriched in genes only found in the four other *Aureobasidium* genomes (adj. p-value < 0.001).

Overall, the genome of *A. pullulans* NBB 7.2.1 and of all five *Aureobasidium* species included here are comparable in size and gene model count and harbour a number of genes and gene clusters that may contribute to biocontrol activity. For example, hydrolase genes with a predicted signal peptide, and in particular esterases, glycosylases, and peptidases, were strongly overrepresented. All of these *Aureobasidium* species thus likely secrete a plethora of hydrolytic enzymes into their surroundings, which might represent one of the mechanisms conferring antagonistic activity to these species. Based on our genome analysis, *A. pullulans* NBB 7.2.1 is predicted to secrete 248 hydrolytic enzymes (including 39 genes encoding predicted secreted proteases) and to produce yet unidentified secondary metabolites as indicated by the 25 secondary metabolite clusters that were identified and which comprise 366 genes overall.

### Transcriptome analysis of *A. pullulans* NBB 7.2.1 competing with *F. oxysporum* NRRL 26381/CL57

In order to identify genes that may be responsible for the biocontrol activity detected in *A. pullulans* NBB 7.2.1, the yeast-like fungus was added to a *F. oxysporum* NRRL 26381/CL57 liquid culture that had been growing for two days and the transcriptomes were analysed by dual RNA-seq [45]. The addition of *A. pullulans* strongly inhibited the growth of *F. oxysproum*, as observed on agar plates. Gene expression was quantified by mapping sequence reads to the high-quality *A. pullulans* NBB 7.2.1 reference genome and the published *F. oxysporum* NRRL 26381/CL57 genome [46]. Since it was the aim of this study to identify genes potentially conferring biocontrol activity, we focused this analysis on *A. pullulans* NBB 7.2.1 genes that were upregulated following the encounter with the plant pathogen *F. oxysporum*.

#### Overall results

**Figure 4:**
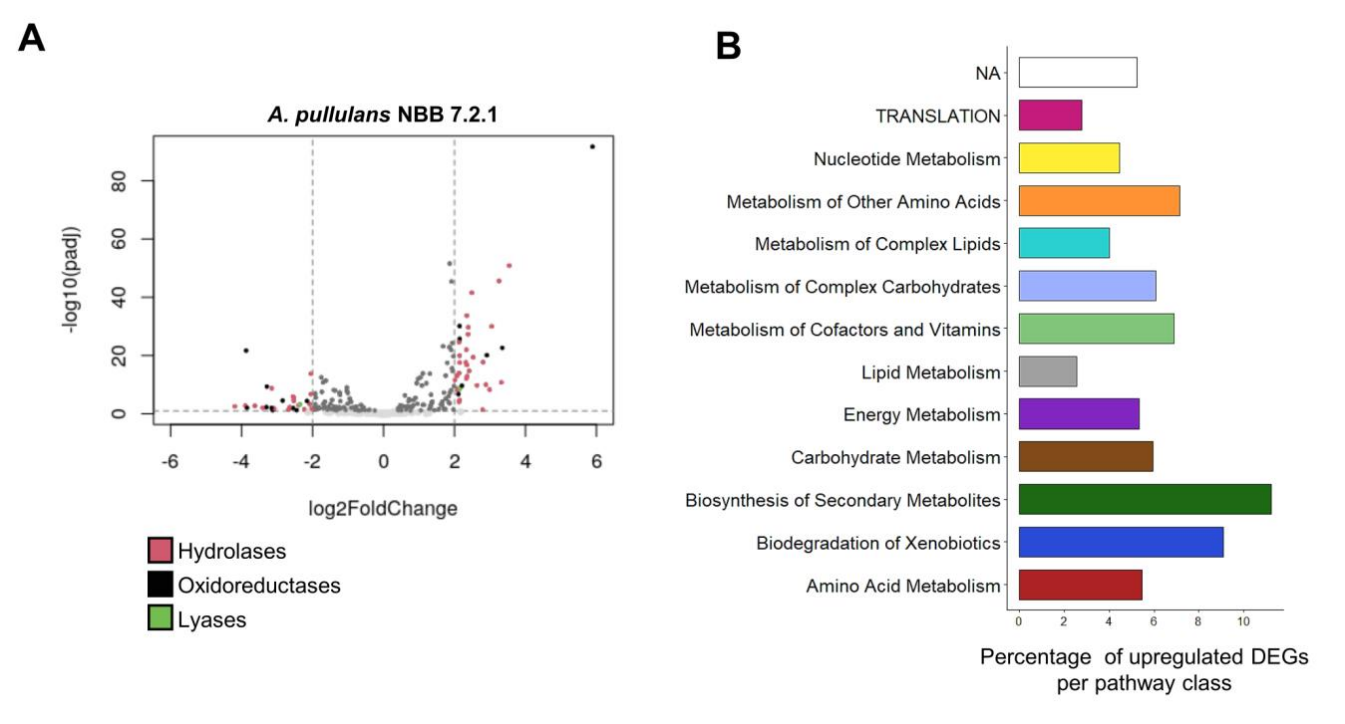
FIGURE 4: Transcriptome analysis of *A. pullulans* NBB 7.2.1 and *F. oxysporum* NRRL 26381/CL57 in pure culture and during competition with each other. *A. pullulans* NBB 7.2.1 strongly responds to co-cultivation with *F. oxysporum* NRRL 26381/CL57 at the transcriptome level. **(A)** Volcano plot showing expression of the *A. pullulans* NBB 7.2.1 genes with a signal peptide. In the co-culture, 178 genes exhibited significantly changed expression by at least a factor of four (92 and 86 up- and downregulated genes, respectively) compared to pure culture. **(B)** Proportion of upregulated DEGs (log2FoldChange > 2) for genes annotated to different KEGG categories. Biosynthesis of secondary metabolites (11.2%), biodegradation of xenobiotics (9.1%), metabolism of other amino acids (7.1 %), and metabolism of cofactors and vitamins (6.9 %) comprised the categories with the highest frequency of upregulated genes.

Nearly 25 million raw reads per sample were trimmed and filtered, which resulted in over 93% high quality reads per sample, that were included for further analyses (Supplementary Table 7). More than 96.9% of all reads had a base call accuracy of at least 99% and the respective replicates clustered together in a PCA analysis of the log transformed counts. Between 91 and 93% of the reads in the co-culture treatment were mapped to the genome of *F. oxysporum* NRRL 2638/CL57, while 7-9% corresponded to expressed *A. pullulans* NBB 7.2.1 genes. This can be explained by the larger amount of biomass filamentous fungi produce as compared to yeasts and the two-day preculture of *F. oxy-sporum* in the liquid medium. After controlling for quality (p-value <0.05), 10,842 genes from *A. pullulans* and 17,466 from *F. oxysporum* were included in the respective differential expression analyses. Even though fewer reads were mapped to the *A. pullulans* NBB 7.2.1 genome, this yeast-like fungus showed a strong transcriptional response to the presence of *F. oxysporum* NRRL 26381/CL57. Overall, 1,337 *A. pullulans* NBB 7.2.1 genes (12% of all genes) were differentially expressed (log2FoldChange>2, p<0.05; 618 and 719 up- and downregulated genes, respectively) as a response to the interaction with *F. oxysporum* NRRL 26381/CL57 (Supplementary Figure S2A). Among the 1,044 genes containing a predicted signal peptide, 178 (17%) showed significantly changed expression (92 and 86 up- and downregulated genes, respectively), significantly more than in the genes without a predicted signal peptide (p<0.01; **Figure 4A**). The *A. pullulans* NBB 7.2.1 genes most strongly upregulated in the presence of *F. oxysporum* NRRL 26381/CL57 comprised a large number of uncharacterised genes (annotated as hypothetical genes; i.e., 33 of the 50 most upregulated genes). Among the genes with an annotation, the KEGG categories “biosynthesis of secondary metabolites” (11.2%), “biodegradation of xenobiotics” (9.1%), “metabolism of other amino acids” (7.1 %), and “metabolism of cofactors and vitamins” (6.9 %) comprised the highest frequency of up-regulated genes (**Figure 4B**). Despite the large number of sequencing reads, we detected only a weak response of *F. oxysporum* NRRL 26381/CL57 to the competition with *A. pullulans* NBB 7.2.1 (**Figure 2B** and **2C**). Only 80 genes were differentially expressed: 36 were upregulated and 44 downregulated. Few of these 80 DEGs (differently expressed genes) have a functional description in the *F. oxysporum* NRRL 26381/CL57 genome annotation as 53 genes were annotated to encode hypothetical proteins. Upregulated *F. oxysporum* NRRL 26381/CL57 DEGs mainly belonged to the KEGG categories “metabolism of complex lipids”, “lipid metabolism” and “carbohydrate metabolism” (Supplementary Figure 2D). Only two upregulated DEGs, one of which was a putative α-galactosidase, had a predicted signal peptide. An aldehyde dehydrogenase (oxidoreductase), two α-galactosidases, a p-aminomuconate deaminase (hydrolase), and a lactoylglutathione lyase were upregulated as well. Four additional, weakly upregulated genes, a β-N-acetylhexosaminidase (3.4 fold-change (FC)), a chitin deacetylase (2.1 FC), a monophenol monooxygenase (1.5 FC) and an alkaline endopeptidase (Oryzin; 1.3 FC), contained a signal peptide and thus likely represent secreted proteins.

#### Genes involved in secondary metabolite production

**Figure 5:**
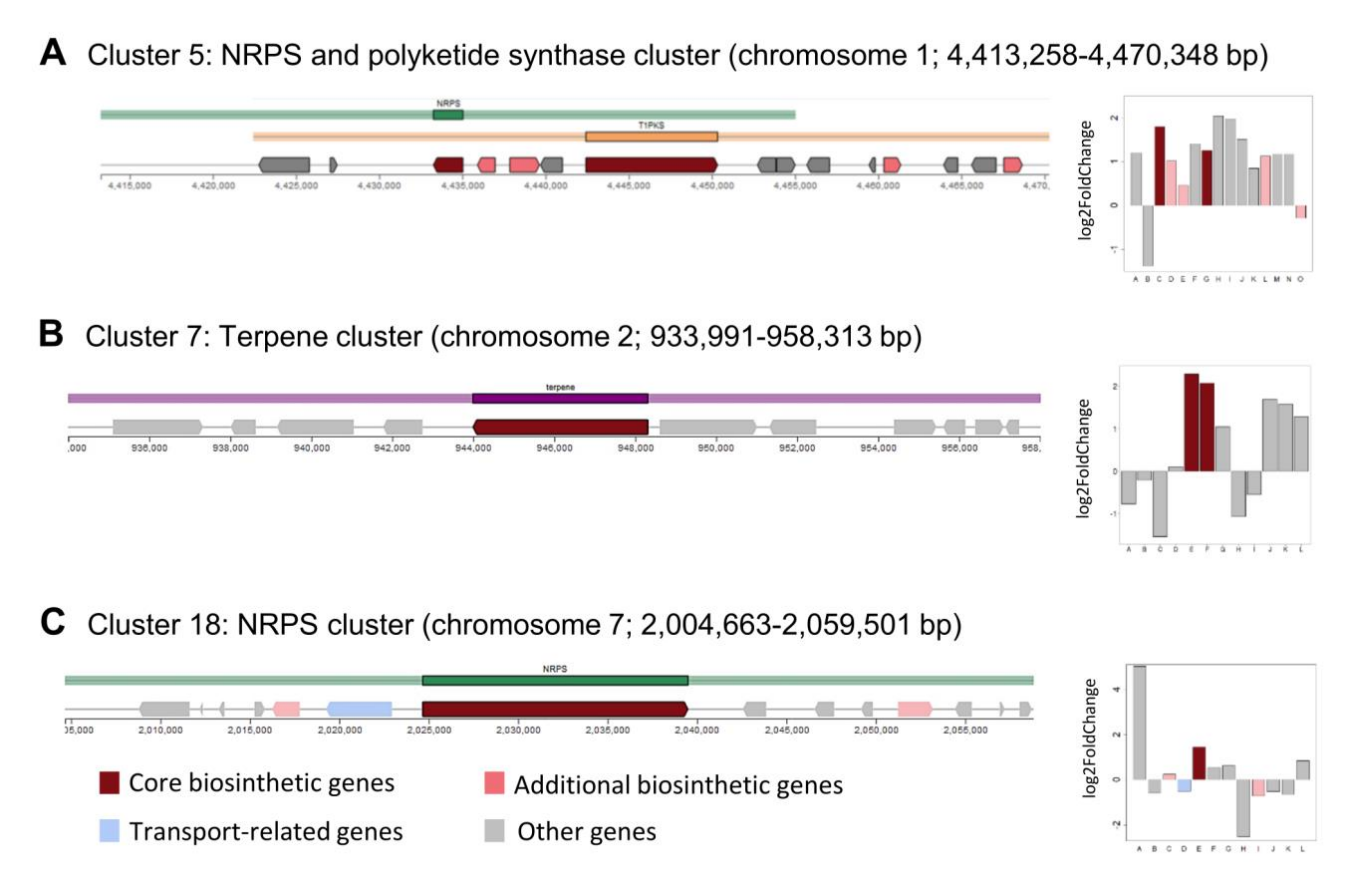
FIGURE 5: Identification of secondary metabolite clusters in the *A. pullulans* NBB 7.2.1 genome with the fungal antiSMASH v.5.1.2 online tool [41, 42]. Core biosynthetic genes of three *A. pullulans* NBB 7.2.1 secondary metabolite clusters were upregulated in response to co-cultuvation with *F. oxysporum* NRRL 26381/CL57. The core biosynthetic genes (dark red colour) of cluster 5 (NRPS and polyketide synthase cluster) **(A)**, 7 (terpene cluster) **(B)**, and 18 (NRPS cluster) **(C)** were upregulated after one day of interaction with *F. oxysporum* NRRL 26381/CL57.

Overall, more than 10% of the genes in the KEGG category “biosynthesis of secondary metabolites” were upregulated (**Figure 4B**). These 15 DEGs belonged to the flavonoid (8), alkaloid type I (3) and erythromycin biosynthesis (3) pathways. Biosynthetic enzymes from three of the antiSMASH clusters identified in the *A. pullulans* NBB 7.2.1 genome (cluster 5, 7, and 18; see above) showed increased expression during the competition with *F. oxysporum* NRRL 26381/CL57. Two gene products from the NRPS (Protein ID 34549) and polyketide synthase (Protein ID 78529) cluster 5 were upregulated with a log2 FC of 1.79 and 1.25, respectively (**Figure 5A**). The putative core biosynthetic gene in the terpene cluster 7 was predicted as two different genes in our independent genome annotation and subsequent transcriptome analysis (Protein ID 37339 and 39349; **Figure 5B**). Both of these genes were strongly upregulated (log 2 FC of 2.29 and 2.07). Four other genes in the same cluster were upregulated with a log2 FC above 1. In cluster 18, a NRPS gene (Protein ID 49318) was upregulated by a log2 FC of 1.4 (**Figure 5C**).

#### Genes with a predicted signal peptide

**Figure 6:**
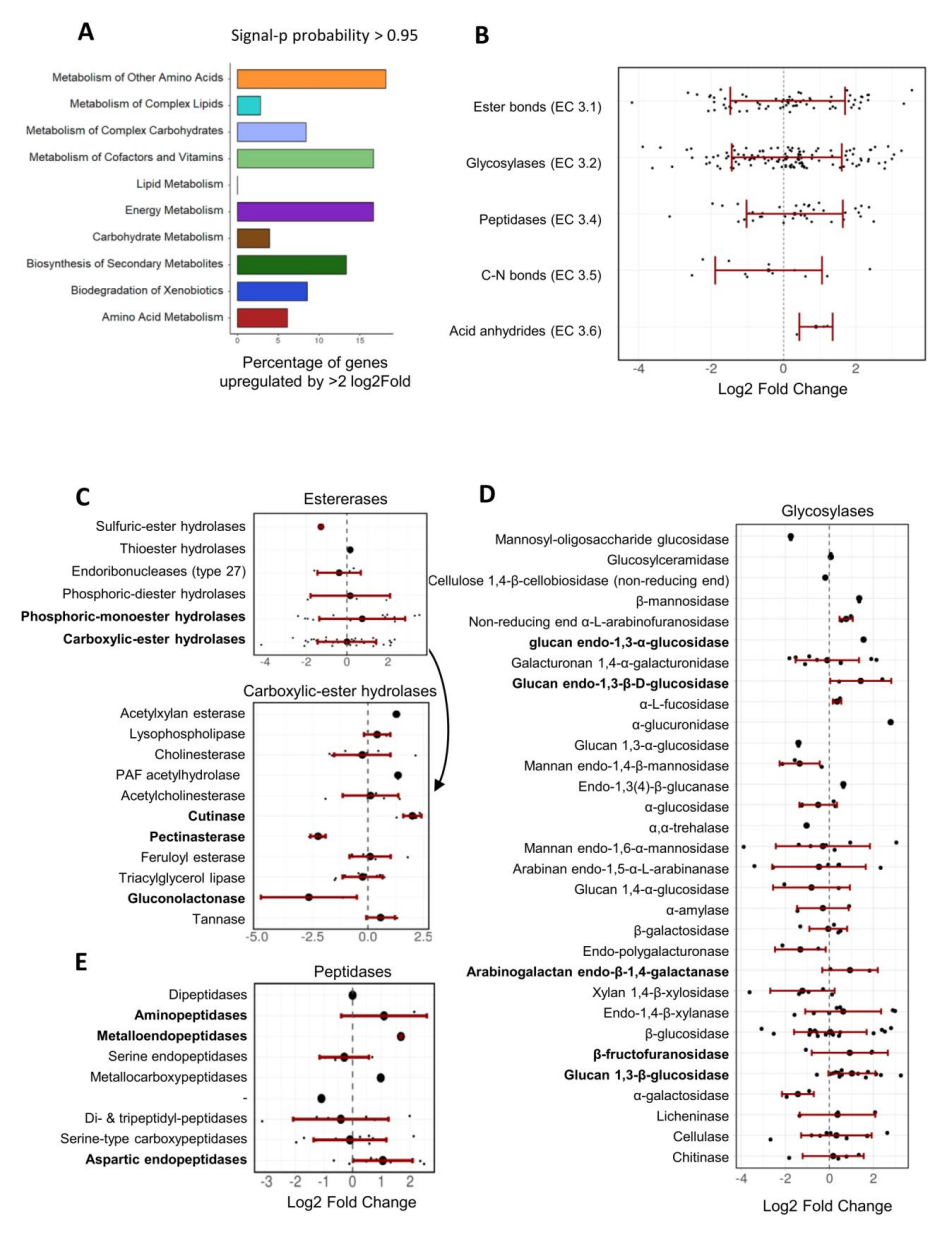
FIGURE 6: Transcriptional regulation of *A. pullulans* NBB 7.2.1 genes with a predicted signal peptide upon co-cultivation with *F. oxysporum* NRRL 26381/CL57. Many *A. pullulans* NBB 7.2.1 hydrolase genes were strongly up- or downregulated during the interaction with *F. oxysporum* NRRL 26381/CL57. **(A)** Percentage of upregulated DEGs in each KEGG pathway class for those *A. pullulans* NBB 7.2.1 genes containing a predicted signal peptide. **(B)** Variation of the Log2 FC values within each hydrolase enzyme category. Variation of the Log2 FC values of all *A. pullulans* NBB 7.2.1 DEGs is also shown on a lower level of categorisation for hydrolases acting on ester bonds **(C)**, glycosylases **(D)**, and peptidases **(E)**.

For the genes with a signal peptide sequence, “metabolism of other amino acids”, “metabolism of cofactors and vitamins”, and “energy metabolism” were the KEGG pathways comprising the highest proportion of upregulated genes (approx. 15% of the genes in each of these three categories; **Figure 6A**). On average, hydrolase genes were not significantly up- or down-regulated, but in particular those encoding predicted esterases (EC 3.1), glycosylases (EC 3.2) and peptidases (EC 3.4) comprised numerous genes that were strongly upregulated in the presence of *F. oxysporum* NRRL 26381/CL57 (**Figure 6B**). All three hydrolase genes predicted to encode enzymes acting on acid anhydrides were up-regulated. Within the esterases, some of the genes annotated to encode phosphoric-monoester hydrolases were the most strongly upregulated, while predicted carboxylic-ester hydrolases (in particular pectinesterases and gluconolactonases) comprised the most strongly downregulated genes (**Figure 6C**). In contrast, all three genes predicted to encode cutinases (also belonging to the carboxylic-ester hydrolases), were strongly upregulated in the competition with *F. oxysporum* NRRL 26381/CL57. Among the predicted glycosylase genes, those encoding β-fructofuranidases, glucan endo-1,3-β-D-glucosidases, glucan 1,3-β-glucosidases, glucan endo-1,3-α-glucosidases, and arabinogalactan endo-β-1,4-galactanases were mostly up-regulated, but the majority of these categories only comprised few genes (**Figure 6D**). The peptidases comprised three categories that showed at least a two-fold upregulation on average; namely the metalloendopeptidases, aspartic endopeptidases, and aminopeptidases (**Figure 6E**). Finally, lyases with a predicted signal peptide (ten genes) were enriched in the *A. pullulans* NBB 7.2.1 genome (see **Figure 3C**). Three of these lyase genes were upregulated, while two were downregulated (Supplementary Figure 2E). The three upreglated genes comprised a carbonic anhydrase, upregulated by over 4-fold, and two pectate lyases that were upregulated by more than 2.5-fold. However, a third pectate lyase gene was strongly downregulated.

Overall, this dual RNA-seq experiment confirmed the transcription and upregulation of 67 of the 1129 potential *A. pullulans* NBB 7.2.1 biocontrol genes (i.e., secreted hydrolase genes and genes comprised in secondary metabolite synthesis) during the competition with *F. oxysporum* NRRL 26381/CL57. The functional category “biosynthesis of secondary metabolites” comprised the highest frequency of upregulated genes and specific groups of putatively secreted hydrolases, such as acid anhydrases, phosphoric-monoester hydrolases, cutinases, specific glucosidases and aminopeptidases and endopeptidases, comprised strongly upregulated genes. We thus hypothesise that these genes contribute to the complex biocontrol phenotype of *A. pullulans* NBB 7.2.1.

### *A. pullulans* NBB 7.2.1 secretome analysis during competition with *F. oxysporum* NRRL 26381/CL57

To complement our dual RNA-seq approach and to directly analyse secreted proteins that may mediate biocontrol activity, we collected and enriched proteins secreted by *A. pullulans* NBB 7.2.1, by *F. oxysporum* NRRL 26381/CL57, and during the interaction of both fungi under the same experimental conditions used for the transcriptome analysis. Proteins were identified by searching tandem mass spectrometry data against the protein databases based on the reference genomes of *A. pullulans* NBB 7.2.1 and *F. oxysporum* NRRL 26381/CL57, respectively. Overall, and as expected, our differential secretome analysis of *A. pullulans* NBB 7.2.1 culture supernatants did not correlate well with the transcriptome results. This effect was expected as it had been similarly reported in other systems [47–52]. In general, protein and transcriptome levels do not seem to be highly correlated [53], which is likely due to the additional levels of regulation that determine protein levels (i.e., translation, post-translational modification, formation of complexes, degradation). Importantly, the analytical methods used to quantify transcript and protein levels have vastly different sensitivities and result in different coverage, thus complicating the comparison of the two. Transcriptome and secretome analyses are thus complimentary approaches.

**Figure 7:**
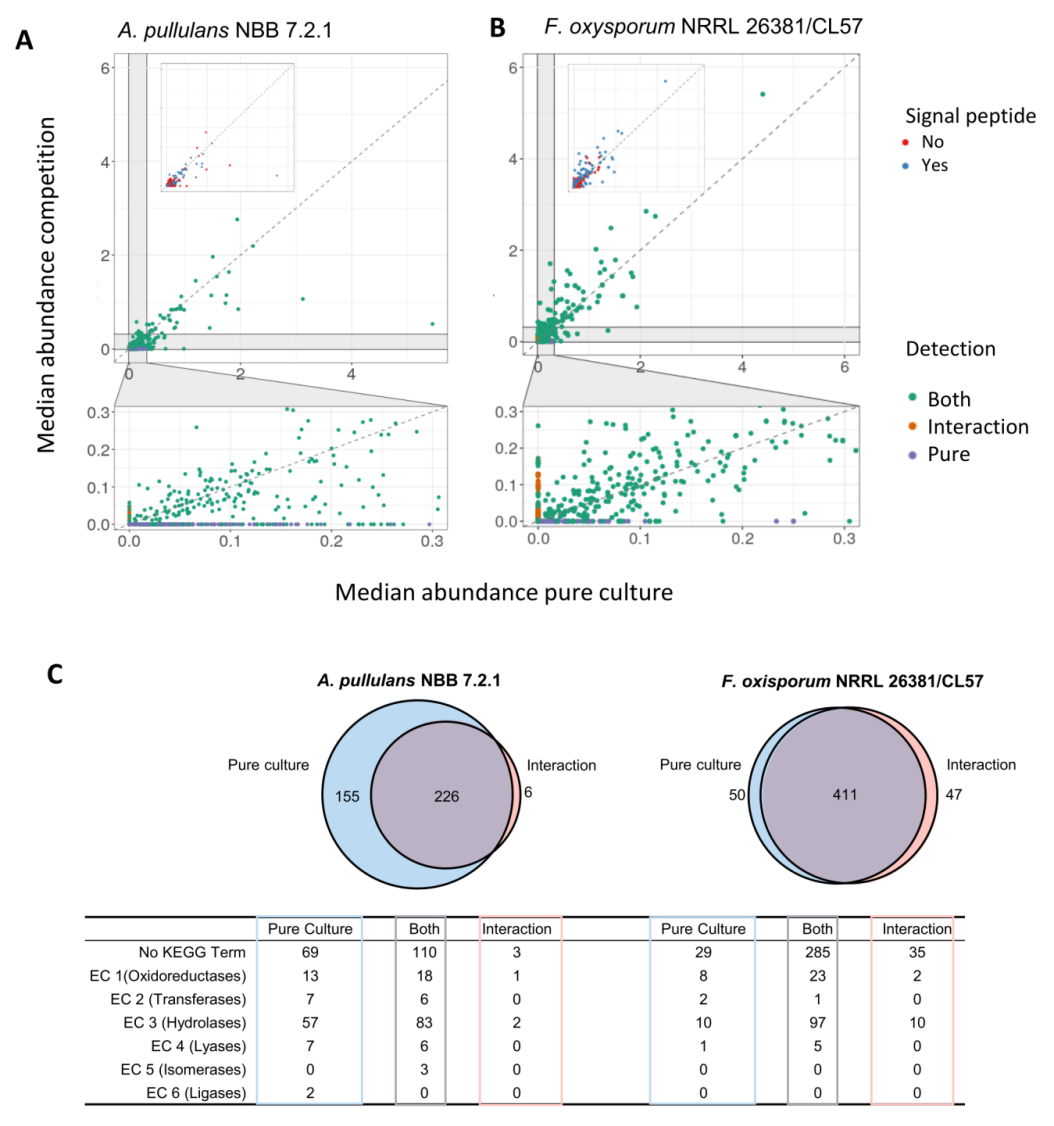
FIGURE 7: Secretome analysis of *A. pullulans* NBB 7.2.1 and *F. oxysporum* NRRL 26381/CL57 pure cultures and a co-culture. Supernatant from pure and co-cultures of *A. pullulans* NBB 7.2.1 and *F. oxysporum* NRRL 26381/CL57 were filtered with a 0.2 µm membrane and concentrated with > 50 kDa ultrafiltration tubes. These extracts of secreted proteins were analysed with a proteomics pipeline. Median abundance of all proteins detected in pure culture (x-axis) and during the competition (y-axis) for *A. pullulans* NBB 7.2.1 **(A)** and *F. oxysporum* NRRL 26381/CL57 **(B)**. In the upper panel insert, proteins with or without a predicted signal peptide are highlighted in blue and orange color, respectively. The lower panel shows proteins of low abundance and highlights those only detected during the competition (orange) or in pure culture (blue). **(C)** For *A. pullulans* NBB 7.2.1, only few proteins were uniquely found during the competition with *F. oxysporum* NRRL 26381/CL57, while for *F. oxysporum* NRRL 26381/CL57 almost equal numbers of unique proteins were detected in the pure culture and the competition. KEGG term annotation of the proteins in the different categories is indicated.

Few *A. pullulans* NBB 7.2.1 and *F. oxysporum* NRRL 26381/CL57 proteins were highly abundant, while the vast majority was detected in comparable amounts and at relatively low abundance (**Figures 7A** and **7B**). The most abundant proteins in the *A. pullulans* NBB 7.2.1 pure culture and during the competition largely overlapped: 50 proteins were abundant in both treatments, 24 only in the pure culture, and eleven only in the interaction experiment. This was also true for *F. oxysporum* NRRL 26381/CL57 proteins (110 proteins were abundant in both treatments and 36 and 21 only in the interaction or the pure culture, respectively).

Since most proteins had a low abundance and their expression varied among the four replicates, we filtered out proteins detected only in one sample (i.e., we only analysed further those proteins with a median abundance higher than 0). For *A. pullulans* NBB 7.2.1, this identified a set of 381 and 232 proteins in the pure culture and the interaction, respectively (**Figure 7C**). Of these proteins, 226 were identified under both conditions, 155 were only detected in the pure culture, while six proteins were found only in the interaction experiment. Among the 381 proteins detected in the supernatant of the pure *A. pullulans* NBB 7.2.1 culture and in comparison to those proteins not detected in the secreted proteome, sequences containing a predicted signal peptide were significantly overrepresented (p-value<0.001, 45% of all proteins in the secreted proteome, 171 proteins). The 381 proteins were also significantly enriched in hydrolases (EC 3, adj. p-value<0.001, 140 proteins), and lyases (EC 4, adj. p-value<0.008, 13 proteins). Similarly, the 232 *A. pullulans* NBB 7.2.1 proteins detected during the interaction with *F. oxysporum* NRRL 26381/CL57 were significantly enriched in proteins with a predicted signal peptide (p-value<0.001, 58% or 134 proteins) and in predicted hydrolases (EC 3, adj. p-value<0.001, 85 proteins). The six proteins detected in the interaction alone comprised two hydrolases (a mannan endo-1,4-β-mannosidase (EC 3.2.1.78) and a thiolester hydrolase (EC 3.1.2)), as well as an oxidoreductase (alcohol dehydrogenase, EC 1.1.1.1), and three proteins without a KEGG annotation. Interestingly, this alcohol dehydrogenase, a predicted GroES-like chaperonin, was upregulated by 1.74 log2 FC in the transcriptome. To detect proteins with a higher abundance in the interaction, we used the set of 226 proteins present in both treatments and calculated the FC of the median. Overall, we found 32 *A. pullulans* NBB 7.2.1 proteins with a FC higher than 1.5 (Supplementary Table 8). These 32 proteins comprised twelve hydrolases (EC 3), a oxidoreductase (superoxide dismutase, EC 1.15.1.1), and 19 proteins lacking a KEGG annotation. More specifically, the hydrolases comprised two esterases (feruloyl esterase, EC 3.1.1.73; serine/threonine-specific protein phosphatase, EC 3.1.3.16), five glycosylases (α-manno-sidase, EC 3.2.1.24; β-galactosidase, EC 3.2.1.23; α-gluco-sidase, EC 3.2.1.20; and two β-glucosidases, EC 3.2.1.21), two proteases (glutamate carboxypeptidase II, EC 3.4.17.21; aminopeptidase I, EC 3.4.11.22) and three amidases (β-ureidopropionase EC 3.5.1.6; formamidase EC 3.5.1.49; dihydroorotase EC 3.5.2.3).

In the *F. oxysporum* NRRL 26381/CL57 secretome, 461 and 458 proteins were detected in the pure culture and the interaction, respectively (50 only in the pure culture, 411 under both conditions, and 47 only during the interaction). The latter group was composed of 41 proteins with no KEGG annotation and six hydrolases. These hydrolases included four glycosylases, an α-L-arabinofuranosidase (EC 3.2.1.55), a β-glucosidase (EC 3.2.1.21), a cellulase (EC 3.2.1.4) and a β-fructofuranosidase (EC 3.2.1.26) and two peptidases; deuterolysin (EC 3.4.24.39) and a metallocarboxypeptidase (EC 3.4.17). The interaction with *A. pullulans* NBB 7.2.1 resulted in the upregulation of 38 genes. Two of these were choline dehydrogenase oxidoreductases (EC 1.1.99.1), one a xyloglucan:xyloglucosyl transferase (EC 2.4.1.207), 16 hydrolases and 18 proteins without a KEGG annotation.

Overall, this secretome analysis identified almost 900 secreted *A. pullulans* NBB 7.2.1 and *F. oxysporum* NRRL 26381/CL57 proteins. 38 *A. pullulans* NBB 7.2.1 proteins (32 upregulated and six only detected during the interaction), were identified as interesting targets for further analyses. Among these 38 proteins, predicted hydrolases were enriched within the *A. pullulans* NBB 7.2.1 secretome. Four of these *A. pullulans* NBB 7.2.1 hydrolases were only found during the interaction with *F. oxysporum* NRRL 26381/CL57 or strongly upregulated under this condition, while another eleven had a higher abundance during the interaction, marking these proteins as interesting targets for functional analyses.

## DISCUSSION

The species *A. pullulans* is highly abundant worldwide and thrives in vastly different types of temperate and extreme environments, which may also be the underlying reason for its success as a biocontrol organism. The study presented here reports a reference genome, transcriptome datasets, and a secretome analysis of the *A. pullulans* strain NBB 7.2.1, which strongly inhibits the growth of filamentous fungi. This reference biocontrol strain was compared with four other *Aureobasidium* strains that were studied with respect to their biotechnological potential, stress tolerance, and speciation. Considering the vast number of *Aureobasidium* strains that are reported to strongly inhibit plant pathogenic fungi, it is not surprising that the five strains compared here all exhibited strong antagonistic activity against *F. oxysporum in vitro* (**Figure 1**). Only when diluted, the reproducible and slightly stronger inhibitory activity of *A. pullulans* EXF-150 and NBB 7.2.1, in comparison to the other three strains (*A. melanogenum* CBS 110374, *A. namibiae* CBS 147.97, and *A. subglaciale* EXF-2481), which are not reported as biocontrol organisms, could be detected. This difference in antagonistic activity was not due to differences in growth or cell density, but growth morphology did differ and may explain the weaker antagonism exhibited by *A. subglaciale* EXF-2481. The closer similarity of the two *A. pullulans* isolates was also highlighted by the pangenome analysis, but less apparent based on the functional annotations. This might have been caused by differing KEGG database versions (even though the number of functionally annotated genes was comparable for all five genomes) and the fact that the majority of predicted genes do not have a functional annotation. Overall, these results confirmed the strong biocontrol activity of *A. pullulans* and were the motivation to identify candidate biocontrol genes that confer this activity.

Secondary metabolites and secreted proteins have biotechnological potential and were proposed to be involved in plant-protection and antagonistic effects of *A. pullulans* against plant pathogens [8]. As in other *Aureobasidium* isolates, a large number of secondary metabolite clusters were identified in strain NBB 7.2.1 by the fungal antiSMASH tool. This resource integrates a wealth of different tools for the identification of secondary metabolites and searches a database containing all known secondary metabolite clusters by aligning genome regions [41, 42]. Since antiSMASH searches a database specifically designed for the identification of secondary metabolite clusters, and does not rely on general gene annotation databases (e.g., COG, KOG, KEGG, Pfam, Interpro), this tool identified many more genes potentially involved in the production of secondary metabolites. Interestingly, 15 clusters containing NRPS or NRPS-like synthases were predicted for this isolate, while the genomes of *A. melanogenum* and *A. namibiae* encode only seven and nine such clusters, respectively (Supplementary Table 4). NRPS and NRPS-like synthases are large multi-domain enzymes that assemble peptides without mRNAs or ribosomes. In bacteria, these genes often reside in repeats. Comparison of a complete *de novo* assembled *Pseudomonas aeruginosa* MPAO1 genome with ten widely used PAO1 lab strains revealed that 50% or more of the NRPS genes could be missed in their fragmented Illumina short read-based assemblies [54]. Though structurally and functionally diverse, many peptides synthesised by NRPS have been described as secreted antimicrobial agents and toxins [55]. Melanin is known to have a protective role for fungi under various stress conditions and is well described as a product of *A. pullulans* [2]. Neither the melanin PKS cluster, nor other genes predicted to be involved in melanin biosynthesis showed significant expression changes. Thus, the production of melanin does not seem to play a role in the biocontrol activity of *A. pullulans*, but rather an adaptation to a wide variety of environments. Based on our analyses, we conclude that the *A. pullulans* NBB 7.2.1 genome harbours diverse secondary metabolite biosynthesis genes and gene clusters that are yet uncharacterised. The strong upregulation of some of these genes during competition with *F. oxysporum,* in particular of several genes of the terpene cluster, may suggest a role in biocontrol activity, but more detailed molecular studies (e.g., overexpression and deletion, biochemical analyses) are required to determine the exact functions of these genes.

Hydrolases have been repeatedly implicated in yeast biocontrol activity. The best characterised examples are exoglucanases from *Candida oleophila* that have been studied at the molecular level [56–58]. In the *A. pullulans* strain PL5, an alkaline serine protease was described and characterised [18, 19]. The corresponding gene in our *A. pullulans* NBB 7.2.1 genome was slightly and strongly downregulated at the transcriptional and protein level, respectively. However, the comprehensive analysis presented here revealed insights into the large diversity of hydrolytic enzymes *A. pullulans* can secrete into the environment, including proteinases, glycosylases and esterases. This medley underlines the potential of the genus *Aureobasidium* for metabolite and enzyme discovery for biotechnological applications. In the *A. pullulans* NBB 7.2.1 genome, we observed a genomic over-representation of hydrolases potentially involved in the degradation of fungal cell walls and plant products (e.g., glycosidases, proteases, pectate and pectin lyases, as well as cellulases, xylanases, cutinases, and fructofuranosidases). Some of these enzymes were transcriptionally upregulated upon exposure of *A. pullulans* NBB 7.2.1 to the plant pathogen *F. oxy-sporum*. Among the enzymes targeting ester bonds, we found a strong upregulation of predicted cutinases. Other plant-degrading enzyme groups, such as cellulases, polygalacturonases or pectinesterases, were downregulated or their transcript levels remained unaffected. Though the upregulation of potentially plant degrading enzymes was unexpected, we believe this result substantiates the epiphytic ecology of the *A. pullulans* NBB 7.2.1 isolate, as compared to other isolates from glacial and desert habitats, and might be related to the ecological complexity of the plant microenvironment. It should also be emphasised that none of these enzymes have been characterised biochemically. A gene in *A. pullulans* NBB 7.2.1 may show sequence similarity to plant cuticle targeting enzymes, but the natural target of these enzymes is unknown and could also be a microbial component.

Enzymes targeting peptide bonds are implicated in many microbial interactions and are often identified as virulence factors [59–62]. In *A. pullulans*, proteinases have already been identified, but their role in biocontrol activity is not clear. Aminopeptidases can also play roles in co- or post-translational modifications, removing residues from the N-terminus, and producing proteoforms that may affect protein activity. Furthermore, they can degrade small peptides or target specific proteins in order to generate active peptides or inactivate inhibitory proteins [63–66]. The genome and transcriptome analysis of *A. pullulans* NBB 7.2.1 identified specific classes of peptidases that were overrepresented or whose transcripts where up-regulated in *A. pullulans* cells exposed to the filamentous fungus *F. oxysporum*. Aspartic peptidases were enriched in the genomes of *A. pullulans* EXF-150 and NBB 7.2.1 strains. Our transcriptome analysis revealed an increase in the transcription of six aspartic endopeptidases, which use a water molecule activated by two aspartic residues to break the scissile bond. Four enzymes were defined as Aspergillopepsins by our KEGG analyses, while the others were similar to a Candidapepsin and a Yapsin. Aspergillopepsins are known secreted proteinases from the genus *Aspergillus* and have nutritional relevance in acidic environments and structural targets in a variety of organisms. The metallocarboxypeptidase “Peptidase M28” (ID 49010) was upregulated in the proteome (Supplementary Table 8). Overall, these results identified a plethora of proteinases that are secreted by *A. pullulans* NBB 7.2.1 under different conditions. The expanded number of serine-dependent protease genes and transcriptional upregulation of some proteinases during competition with *F. oxysporum* may indicate that proteinases are, at least in part, responsible for the strong biocontrol activity and competitiveness of this yeast-like fungus. The identified protease genes are thus a promising starting point for deciphering biocontrol activity of *A. pullulans* NBB 7.2.1 at the molecular level.

**Figure 8:**
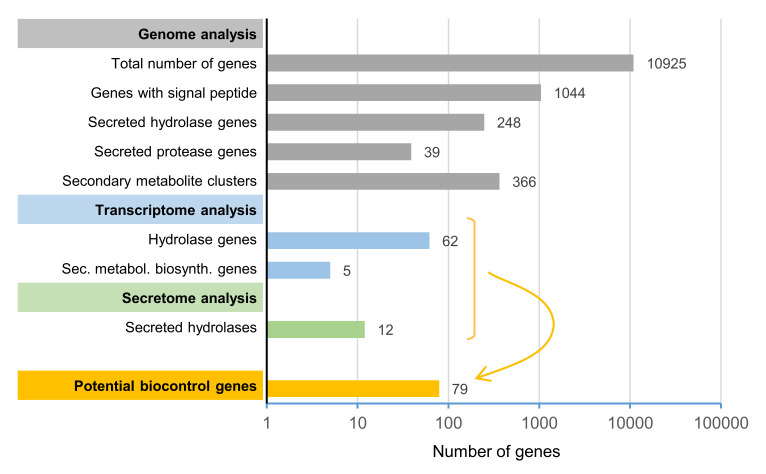
FIGURE 8: Genome, transcriptome, and secretome analyses were used to reduce the number of target biocontrol genes for functional analyses from 10,925 (total number of *A. pullulans* NBB 7.2.1 genes) to 79. As primary candidates for mediating biocontrol activity, genes upregulated during competition, encoding proteins detected in the secretome during the competition, and predicted to encode hydrolase or secondary metabolite biosynthesis genes were defined. The scale of the x-axis is logarithmic.

The current study aimed to identify a limited subset of potential biocontrol factors of the yeast-like fungus *A. pullulans* against *F. oxysporum* by genome, transcriptome and secretome analyses. Based on our high-quality reference genome, this strain is predicted to produce a wide variety of peptidases, glycosylases and esterases and to have the potential to produce a remarkably diverse range of secondary metabolites. Starting from the high-quality reference genome of *A. pullulans* NBB 7.2.1 that contained 10,925 predicted genes, we identified 1,044 genes with a predicted signal peptide (**Figure 8**). 248 of these genes encoded predicted, secreted hydrolases, which included 39 proteases. Furthermore, we identified 25 potential secondary metabolite clusters that comprised, in total, 366 genes. Five biosynthetic genes of three different clusters (in total summing up to 46 genes) were upregulated during the competition. Dual RNA-seq analysis and mapping to our reference genome also identified 62 potentially secreted hydrolases that were upregulated. In the secretome analysis, 38 *A. pullulans* NBB 7.2.1 proteins were upregulated or only detected during the interaction with *F. oxysporum* NRRL 26381/CL57, of which twelve encoded hydrolases. Combining these transcriptome and secretome analyses results in a concise list of 79 potential *A. pullulans* NBB 7.2.1 biocontrol genes encoding predicted hydrolases or secondary metabolite biosynthesis genes that are the main targets for functional characterisation at the molecular level (**Figure 8** and Supplementary Table 8). This approach demonstrates how the integration of genome, transcriptome and protein data allows defining a manageable list of top potential biocontrol genes (less than 1% of all annotated genes). Whether or not these genes are indeed involved in the biocontrol phenotype will have to be verified by gene deletions or overexpression analyses. This genome, transcriptome, and secretome analysis thus lays the basis for future molecular studies to decipher the biocontrol mechanisms employed by *A. pullulans* NBB 7.2.1. We are convinced that the understanding of these mechanisms will allow to optimise and improve biocontrol applications in the future and to explore whether biocontrol mechanisms against different pathogens rely on different or partially overlapping sets of core biocontrol genes.

## MATERIALS AND METHODS

### Strains and cultivation

*A. pullulans* ((de Bary) G. Arnaud) (SH1515060.08FU; isolate NBB 7.2.1, CCOS1008 (Culture Collection of Switzerland)) was isolated from agricultural soil samples [37]. *A. melanogenum* CBS 110374, *A. namibiae* CBS 147.97, *A. pullulans* EXF-150, and *A. subglaciale* EXF-2481 were obtained from the Microbial Culture Collection Ex (http://www.ex-genebank.com) and the CBS-KNAW culture collection (http://www.wi.knaw.nl/collections/). For transcriptome experiments and competition assays, *F. oxysporum* f. sp. *radicis-lycopersici* (Schlecht as emended by Snyder and Hansen) NRRL 26381/CL57 (ARS Culture Collection, USDA) was used. All isolates were maintained on potato dextrose agar (PDA; Becton, Dickinson and Company, Le Pont de Claix, France) plates, grown at 22°C, and transferred to fresh plates weekly. For confrontation experiments, liquid cultures that were grown overnight (for yeast-like fungi) or for 4 d (for *Fusarium*, freshly inoculated from a frozen stock kept at -80°C) at 22°C in potato dextrose broth (PDB; Becton, Dickinson and Company, Le Pont de Claix, France).

### Competition assays on agar plates

Binary competition assays were performed as previously described [37, 67]. *Aureobasidium* cells grown overnight in PDB were collected, washed and resuspended in water (OD_600_ of 0.001-0.1). Cells were spread (15 μl) on PDA plates, 5 μl of a conidial suspension of *F. oxysporum* were inoculated in the center of the plates, and the plates were incubated at 22°C. Before *F. oxysporum* reached the edge of the control plate (plate without *A. pullulans*), growth was quantified with a planimeter (Planix 5, Tamaya Technics Inc., Tokyo, Japan). The average of the relative growth (growth in presence of yeast/growth on control plate) of four replicates was calculated. All assays were repeated at least twice and showed comparable results.

### Genome sequencing and annotation

*A. pullulans* NBB 7.2.1 cells were collected by centrifugation at 8.000 x g for 10 min from 1.5 mL of PDB over-night culture. The pellet was then exposed to two rounds of 2 min freezing with liquid nitrogen and 1 min 95°C water bath in 200 mL of Harju-buffer (2% Triton X-100, 1%SDS, 100 mM NaCL, 10mM Tris-HCL pH 8.0, 1mM EDTA). Genomic DNA was extracted using a phenol/chloroform protocol and Phase Lock Gel^™^ tubes (QuantaBio, Beverly, MA, USA). DNA was precipitated from the aqueous phase with 200 μl 5M ammonium acetate and 1.5 ml ice-cold 100% ethanol and by centrifugation for 15 min at 4°C. After two 70% ethanol washes, the DNA pellet was resuspended in Tris-EDTA buffer. The final concentration was measured with the Qubit™ 3 fluorimeter (Fisher Scientific AG, Reinach, Switzerland) and quality was assessed by agarose gel electrophoresis.

PacBio sequencing was carried out on a Sequel machine (1 SMRT cell) with size selected fragments (BluePippin system) with an average subread length of approximately 10 kb (Table 1). Two 2 x 300 bp Illumina paired end libraries were prepared using the Nextera XT DNA kit and sequenced on a MiSeq (Table 1). A subset of the highest quality reads (“LEADING:3 TRAILING:3 SLIDINGWINDOW:4:15 MINLEN:36”, rq>15) were extracted using trimmomatic (v. 0.36) [68]. Only paired reads were used for further analyses. PloidyNGS (v.3.1.2) [69] was run using the Illumina data to explore the ploidy level of the genome, which was found to be haploid. Computel (v.1.2) [70] was run using the Illumina data to compute the mean telomere length. The number of the telomere pattern “TTAGGG/CCCTAA” was estimated to be 31. PlasmidSpades (v.3.11.1) [71] was run on the Illumina data in order to detect potential evidence for smaller plasmids, which did not give any results. PacBio subreads were filtered with Filtlong (v.0.2.0) and assembled using Flye (v.2.3.3; default parameters, except: estimated genome size of 30 Mb) [72] and with various length cutoffs including 5, 7, 8, 10, 15, 18, 19, 20, 22 and 25 kb. The assemblies resulted in 13-18 contigs each. The filtered subreads were mapped to the polished contigs using graphmap (v.0.5.2) [73] to verify their completeness. All contigs and mapped reads were individually inspected in the Integrative Genomics Viewer (IGV) [74]. The presence of the telomere sequences at both ends of each contig was checked to further verify an end-to-end assembly (i.e., from telomere to telomere). The mitogenome was identified using BLAST [75], the respective contig was start-aligned and reads were mapped to it using graphmap (v.0.5.2) to verify its circularity and completeness, which could be confirmed. The full mitogenome could only be resolved by the assembly using > 5kb subreads and was missed when a higher cutoff was chosen (i.e., all other assemblies). Short contigs were submitted to BLAST [73] and subsequently removed if they appeared spurious. A combination of the assemblies using cutoffs of 7 (for longer contigs) and 25 kb (for resolving repeats) appeared to deliver the best results (plus the >5kb assembly for the complete mitogenome). Both assemblies were individually polished using five Arrow runs. The filtered subreads were mapped to the polished contigs of both assemblies again using graphmap (v.0.5.2) [73] to identify the best result and a combination of contigs from both assemblies was chosen for further processing (Table 2). The contigs were then subjected to another four polishing rounds using Arrow and subreads with a minimum length of 15 kb (in order to allow for a high enough coverage, but maintain resolved repeat regions). The filtered subreads were mapped again to the polished contigs using graphmap (v.0.5.2) [73] to verify their completeness and were individually inspected in the IGV [74]. The number of telomere patterns at both ends of each contig was counted. The contigs were further polished using four Freebayes (v.1.2.0;) [76] runs and the Illumina reads to correct potential small errors (e.g., homopolymer errors). The extensive polishing and manual curation resulted in a total of 12 complete chromosomes and 1 mitogenome. The total genome size is 28,448,966 b (Table 2).

All five *Aureobasidium* genomes in this study have been annotated using the JGI Annotation pipeline and made available via the JGI fungal genome portal MycoCosm [77–79] (https://mycocosm.jgi.doe.gov). Briefly, the JGI pipeline performs feature prediction, starting with CRISPR elements, then non-coding RNAs, and lastly protein coding gene prediction. The final step is the functional annotation of protein coding genes by comparison with protein families, assignment is performed by searches against COG, KOG, KEGG orthology, Pfam, TIGRfam and Interpro databases. RNAseq data have not yet been used to verify the gene annotations. In addition to precomputed Markov clustering of gene families [80], an “*Aureobasidium* pangenome view” was constructed by building single-ortholog clusters using the Pangloss workflow [39, 40]. In short, all genes were searched against each other by BLASTP in order to determine core, singleton and accessory SOCs.

### Comparative genome analyses

Annotation data for *A. pullulans* EXF-150, *A. melanogenum* CBS 110374, *A. namibiae* CBS 147.97, *A. subglaciale* EXF-2481 and *A. pullulans* NBB 7.2.1 was condensed from JGI using R studio (v 4.0.2). Significant enrichment was assessed using Fisher's exact test and p-values were adjusted for multiple testing (method “Benjamini-Hochberg”). Except when stated otherwise, comparisons were made to all genes with terms in any of the five genomes. The packages ggplot2 (v 3.3.1) [81], flextable (v 0.5.10) [82], RColorBrewer (v1.1.2) [83], scales (v 1.1.1) [84], and VennDiagram (v 1.6.20) [85] (e) were used for plotting.

### Co-culture of *A. pullulans* and *F. oxysporum* for RNA extraction

The co-culture experiments were performed in liquid medium with submerged glass beads as previously described [86]. In short, *F. oxysporum* f. sp. *radicis-lycopersici* was precultured in petri dishes containing PDB and a layer of 4 mm glass beads (Fisher Scientific, Reinach, Switzerland). After 2 days, PDB was removed and the plate was washed twice with an indirect stream of sterile water. Independently, overnight liquid cultures of *A. pullulans* NBB 7.2.1 in PBD were pelleted, and cells were washed twice with sterile water. Cells were resuspended to an OD_600_ of 5 in salt buffer (comprised of 1 g/L potassium phosphate monobasic, 0.5 g/L magnesium sulphate anhydrous, 0.1 g/L sodium chloride, and 0.1 g/L calcium chloride anhydrous) supplemented with 2% bacteriological peptone. The *A. pullulans* suspension was applied to the plates with glass beads and *F. oxysposum* and incubated at 22°C. The corresponding controls with *F. oxysporum* grown in glass bead plates and overnight cultures of *A. pullulans* were likewise washed with water and incubated in salt buffer separately. Samples of the interaction between *A. pullulans* NBB 7.2.1 and *F. oxysporum* NRRL 26381, as well as their respective pure cultures, were collected after 24 hours and freeze-dried for RNA extraction, keeping the glass beads for mechanical disruption. Culture supernatants were stored for protein concentration and analysis of secreted proteins.

### Transcriptome sequencing and analysis

Total RNA was extracted with the TRI reagent solution (Fisher Scientific, Reinach, Switzerland) following instructions from the manufacturer. Cells were disrupted mechanically by high-speed agitation with glass beads (2x for 1 min, 1 min on ice in between). Total RNA was quantified with the Qubit system (Fisher Scientific, Reinach, Switzerland) and its quality and purity was assessed by agarose gel electrophoresis. The 2100 Bioanalyzer system was used for quality control to confirm a RIN≥6.5 and a 28S/18S ratio ≥1.0.

RNA sequencing (RNA-seq) was performed using the BGISEQ-500 platform and an oligo-T enriched library at the Beijing Genomics Institute (BGI). All raw reads (2x100 bp) were trimmed, filtered, and adapters were removed using trimmomatic (v.0.39; parameters: SLIDINGWINDOW:4:15 MINLEN:36) [68] (Supplementary Tables 3 and 4). The filtered and paired reads were further processed using sortmerna (v.2.1) [87] in order to remove potential rRNA contamination (parameters: num_alignments: 1, paired_in, ref: all available rRNA databases). The resulting high quality reads (Supplementary Tables 3 and 5, fifth column) were used for further analyses. The *A. pullulans* JGI gff3 annotation file was reformatted to match the style of Ensembl gtf files using sed commands. The filtered high quality reads were mapped to the indexed reference genome of *A. pullulans* or the concatenated and indexed reference genomes of *A. pullulans* and *F. oxysporum* using hisat2 (v.2.1.0) [88]. The alignment files were subsequently split to contain only reads mapping to one of the genomes (Table 4) for the differential expression analysis. Features were counted in R (v.3.6.2) using the package featureCounts [89] and the feature type “gene” and the string “gene_id”. The RNA-seq data are available at NCBI under the BioProject accession PRJNA702246 (https://www.ncbi.nlm.nih.gov/Traces/study/?acc=PRJNA702246). Differential expression analysis was performed in R using the package DESeq2 [90]. Principal component analysis (PCA) was carried out on the log transformed count table. Pairwise comparisons were performed for all treatments. Fold changes (FC) were calculated and p-values were adjusted for multiple comparisons (method “Benjamini-Hochberg”). Genes with a p-value < 0.05 and a log2FoldChange ratio ≥ 2 were defined as differentially expressed genes (DEGs). ReviGo [91] was used to summarize gene onthology terms. Functional analyses of DEGs was performed based on KEGG pathway terms.

### Identification of secreted proteins

Culture supernatant was collected for each sample and sterilized through a 0.2 m filter. Samples were then passed through a 50 kDa cutoff filter (Sartorius AG, Göttingen, Germany) and the protein concentrations were measured with the Qbit protein assay kit (Fisher Scientific AG, Reinach, Switzerland). Peptides were prepared for mass spectrometry using the iST protein digestion and clean-up kit (PreOmics). Briefly, protein samples were denatured in lysis buffer and digested with trypsin for 1 h at 37°C. Resulting peptides were purified and collected as a filtrate. Each resulting sample was dried, resuspended in 3% ACN, 0.1% formic acid and spiked with iRT peptides (Biognosys AG, Schlieren, Switzerland). An aliquot of 2 µL was transferred to autosampler vials for LC-MS/MS analysis on a Q-Exactive HFX mass spectrometer. 12 LC MS/MS samples (supernatants from pure cultures of *A. pullulans* NBB 7.2.1 or *F. oxysporum* f. sp. *radicis-lycopersici* NRRL 26381/CL57, and from the competition of both fungi; four biological replicates each) were processed using our previously published shotgun proteomics data analysis pipeline [92]. Quality control and conversion of raw data was performed using a wrapper function written in R, that executes ThermoRawFileParser [93] and rawDiag [94]. The converted files were then searched against a protein search database of the annotated genomes of the respective organisms: *Aureobasidium pullulans* NBB 7.2.1(10,925 CDS, https://mycocosm.jgi.doe.gov/AurpulNBB1/AurpulNBB1.home.html) and *F. oxysporum* f. sp*. radicis-lycopersici* NRRL 26381/CL57 (12,103 CDS; Genbank acc. AGNB00000000), to each of which 256 sequences of common contaminants were added. Protein searches were carried out with MS-GF+ (v2020.03.14) [88] using a precursor mass accuracy of 10 ppm, cysteine carbamidomethylation as fixed, and oxidation of methionine as variable modifications. The false discovery rate (FDR) was estimated using the target-decoy approach of MS-GF+. The FDR at the peptide-spectrum-matching (PSM) level was set such that the FDR at the protein-level was below 1%. For protein inference, we only considered proteins which were identified by unambiguous peptides as returned by a PeptideClassifier analysis [95] for *A. pullulans* NBB 7.2.1; for *F. oxysporum*, several peptides could imply more than one protein. In addition, we required at least two distinct peptides or three PSMs for a protein identification under one condition. Protein abundances were calculated as the percentage of total PSMs and filtered by “median > 0”, to exclude proteins detected in only one of the four replicates. Proteins with a median abundance FoldChange ratio ≥ 1.5 were defined as differentially upregulated proteins. Protein abundances were further analysed and visualized using R studio (v 4.0.2). The secretome data are deposited at MassIVE with the dataset identifier MSV000086991 (https://massive.ucsd.edu/ProteoSAFe/dataset.jsp?accession=MSV000086991). Annotation data for *A. pullulans* NBB 7.2.1 and *Fusarium oxysporum* f. sp. *radicis-lycopersici* NRRL 26381/CL57 was used from JGI. Significant enrichment was assessed using the Fisher's exact test and p-values were adjusted for multiple testing (method “Benjamini-Hochberg”). For plotting, the packages ggplot2 (v 3.3.1) [81], RColorBrewer (v1.1.2) [83] and VennDiagram (v 1.6.20) [85] (e) were used.

## SUPPLEMENTAL MATERIAL

Click here for supplemental data file.

All supplemental data for this article are available online at http://www.microbialcell.com/researcharticles/2021a-rueda-mejia-microbial-cell/.

## Abbreviations:

DEG: – differently expressed gene,
FC: – fold change,
NRPS: – non-ribosomal peptide synthase,
PKS: – polyketide synthase,
SOC: – single-ortholog clustering.
