# Supplementary material for: Genome, transcriptome and secretome analyses of the antagonistic, yeast-like fungus Aureobasidium pullulans to identify potential biocontrol genes
Source: Microb Cell. 2021 Jun 8;8(8):184–202. doi: 10.15698/mic2021.08.757 (PMC8329847; doi:10.15698/mic2021.08.757)
Supplement: Supplementary file 1 [file mic-08-184-s01.pdf]

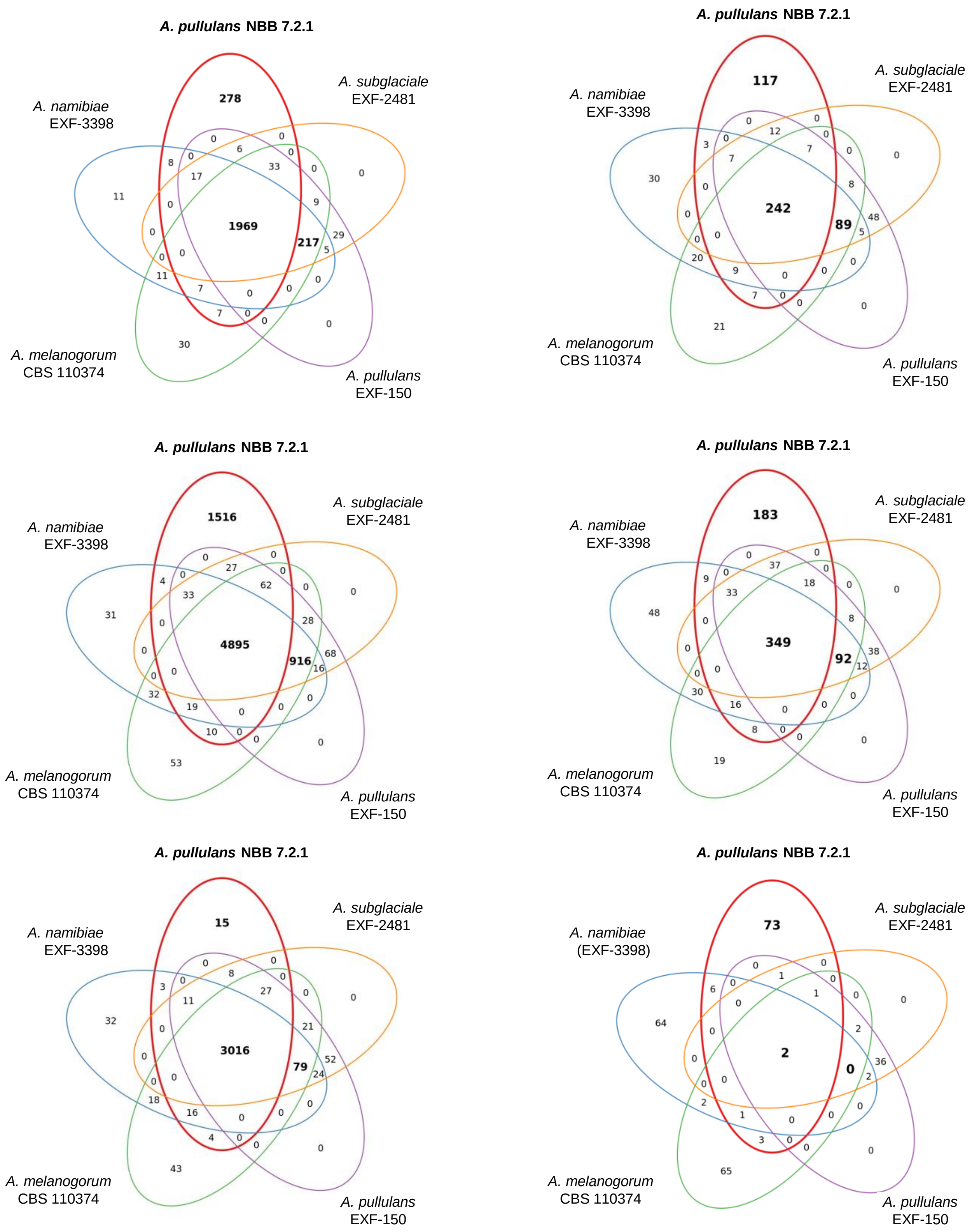

Supplementary Figure S1: Venn diagrams showing the unique term number shared among the five *Aureobasidium* genomes for GO (top), IPR (middle), and KOG terms (bottom). Most terms can be found in every variant (middle part of each Venn diagram). GO- and IPR terms show a substantial number of unique terms found only in *A. pullulans* (NBB 7.2.1) (top part of each Venn diagram) and in all *Aureobasidium* genomes except *A. pullulans* (NBB 7.2.1) (middle right part of each Venn diagram). The results are shown for the entire genome (left) and only those genes containing a predicted signal peptide (right).

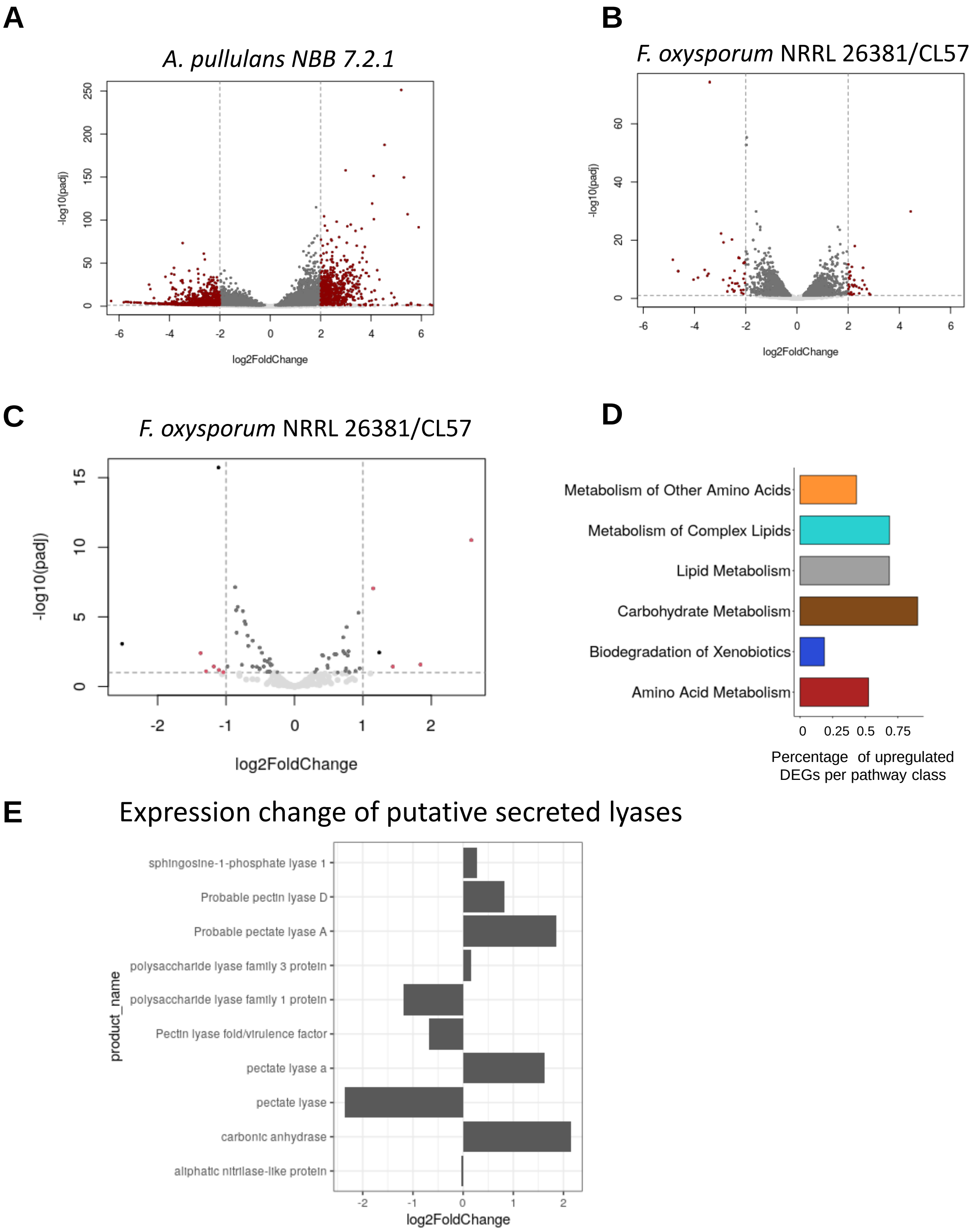

Supplementary Figure 2: Transcriptome analysis of *A. pullulans* NBB 7.2.1 competing with *F. oxysporum* NRRL 26381/CL57. A) Volcano plot for all *A. pullulans* NBB 7.2.1 genes. B) Volcano plot for all *F. oxypsorum* NRRL 26381/CL57 genes. C) Volcano plot for all *F. oxysporum NRRL 26381/CL57* genes with a predicted secretion signal. Only 80 genes were differentially expressed (36 up- and 44 downregulated, respectively. D) Fraction of upregulated DEGs for *F. oxysporum NRRL 26381/CL57* genes annotated to different KEGG categories. These DEGs mainly belonged to the KEGG categories “metabolism of complex lipids”, “lipid metabolism” and “carbohydrate metabolism”. E) Expression changes of the 10 predicted lyase genes with a signal peptide.

Supplementary Table 1: Overview of the *A. pullulans* NBB 7.2.1 genome sequencing data.

Sequencing technology	Coverage	Mean read length	Reads mapped to final assembly [%]
PacBio Sequel, 1 SMRT cell	> 5 kb: 207x<br>> 7 kb: 186x<br>> 15 kb: 130x<br>> 20 kb: 73x<br>> 25 kb: 35x	10,126	98.61
Illumina MiSeq, 2 libraries	29x	2x 300 bp	100

Supplmentary Table 2: Overview of the final *A. pullulans* NBB 7.2.1 genome assembly.

Contigs	Length [bp]	Cov. Pacbio > 15 kb	Cov. Illumina	No. of telomere patterns 5'	No. of telomere patterns 3'	Status
Chromosome I	4'496'211	131	30	16	19	Complete
Chromosome II	3'414'125	130	30	20	26	Complete
Chromosome III	2'798'034	130	29	19	12	Complete
Chromosome IV	2'761'800	130	29	18	16	Complete
Chromosome V	2'389'990	165	29	18	20	Region 1'940–1'990 kb not resolved: 6 consecutive rRNA operons could be resolved, but 15x higher coverage suggests that the actual number is ~100.
Chromosome VI	2'300'035	129	29	11	19	Complete
Chromosome VII	2'219'817	131	29	15	18	Complete
Chromosome VIII	2'042'521	128	28	16	23	Complete
Chromosome IX	1'722'421	127	29	16	18	Complete
Chromosome X	1'577'834	132	29	14	16	Complete
Chromosome XI	1'524'309	130	29	11	18	Complete
Chromosome XII	1'164'306	124	30	24	15	Complete
Mitogenome	37'563	351	128	-	-	Complete, circular
**Whole genome**	**28'448'966**					

Supplementary Table 3: Overview of the genome annotations for the five *Aureobasidium* species from the Mycocosm database.

Species	Predicted Genes	Genes with GO-Terms	Genes wtih KEGG-Terms	Genes with IPR-Terms	Genes with KOG-Terms	Genes with no Term
A. pullulans NBB 7.2.1	10925	5553	2189	7946	5490	2871
A. melanogorum CBS 110374	10594	5731	2345	7027	6306	2699
A. namibiae CBS 147.97	10266	5675	2291	6933	6255	2468
A. pullulans EXF-150	11866	6106	2459	7451	6701	3415
A. subglaciale EXF-2481	10809	5798	2386	7038	6382	2886

Supplementary Table 4: Overview of the secondary metabolite clusters detected in the five *Aureobasidium* genomes.

	A. pullulans NBB 7.2.1	*A.melanogenum* CBS 110374	*A.namibiae* CBS 147.97	*A. pullulans* EXF-150	*A. subglaciale* EXF-2481
NRPS-like	10	5	7	11	8
NRPS	5	2	2	5	5
T1PKS	6	5	5	6	10
Terpene	4	6	3	3	4
ßlactone	2	2	1	2	3
**Total N° of clusters**	**25**	**19**	**18**	**25**	**25**

Supplementary Table 5: Secondary metabolite clusters predicted by antiSMASH (v.5.1.2) in the *A. pullulans* NBB 7.2.1 genome.

Nr	Chr	Location	Number of genes	NRPS/NRPS-like	PKS	Terpene synthase	Betalactone	?
1	1	537,897-581,518	20	✓				
2	1	1,668,707 - 1,696,841	10				✓	
3	1	2,281,375 - 2,324,625	12		✓			
4	1	3,493,232 - 3,514,947	8			✓		
5	1	4,413,258 - 4,470,348	23	✓	✓			
6	2	784,415 - 827,677	18	✓				
7	2	933,911 - 958,313	11			✓		
8	4	2,257,601 - 2,300,729	18	✓				
9	5	1,327,344 - 1,374,410	16	✓			✓	
10	5	1,504,912 - 1,542,012	15	✓				
11	5	1,577,259 - 1,617,443	11	✓				
12	5	1,840,765 - 1,879,046	14	✓				
13	5	2,328,771 - 2,376,201	16	✓				
14	6	1,192,848 - 1,214,008	9			✓		
15	6	1,267,918 - 1,312,990	13	✓				
16	7	914,590 - 955,777	17	✓				
17	7	1,832,570 - 1,874,921	18	✓				
18	7	2,004,633 - 2,059,501	12	✓				
19	8	2,002,411 - 2,042,521	16	✓				
20	9	1 - 25,507	8		✓			
21	9	1,315,225 - 1,361,997	14		✓			
22	9	1,608,846 - 1,652,541	21	✓				
23	10	1,467,446 - 1,515,011	17		✓			
24	12	350,805 - 397,584	21		✓			
25	12	1,071,360 - 1,092,460	8			✓		

Supplementary Table 6: Overview of the number of genes containing a predicted signal peptide in the five *Aureobasidium* genomes from the Mycocosm database. The number of those genes annotated with different descriptive terms is indicated.

	Total Genes	with GO-Terms	wtih KEGG-Terms	with IPR-Terms	with KOG-Terms	with no Term
A. pullulans NBB 7.2.1	1044	501	330	680	95	272
A. melanogorum CBS 110374	816	406	229	478	79	240
A. namibiae CBS 147.97	851	443	244	525	82	219
A. pullulans EXF-150	1013	492	253	576	45	364
A. subglaciale EXF-2481	940	462	243	538	72	299

Supplementary Table 7: Overview of the number of raw, quality trimmed/filtered and rRNA filtered reads for the interaction experiment of *A. pullulans* NBB 7.2.1 (Apul) with *F. oxysporum* NRRL 26381/CL57(Fox).

Sample ID	Treatment	No. of paired raw reads	Trimmed and quality filtered [%]	rRNA filtered [%]	Mapped reads [%]	Mapped to *Apul* [%]	Mapped to *Fox* [%]
B1.1	Apul & Fox	24’762’512	98.8	96.3	96.5	7.0	93.0
B1.3	Apul & Fox	25’906’417	98.7	95.9	97.1	8.9	91.1
B1.4	Apul & Fox	24’922’544	99.0	96.0	97.0	8.5	91.5
B2.2	Apul	24’769’275	98.9	93.5	96.0	100	0
B2.3	Apul	24’543’101	98.6	94.8	95.7	100	0
B2.4	Apul	25’052’566	98.6	94.1	95.7	99.9	0.1
B3.1	Fox	24’942’685	98.6	96.6	97.6	0	100
B3.2	Fox	24’841’985	98.7	96.8	97.6	0	100
B3.4	Fox	24’961’639	98.8	97.3	97.5	0	100

Supplementary Table 8: Overview of the 32 *A. pullulans* NBB 7.2.1 proteins with higher median abundance (FC > 1.5) in the interaction with *F.oxysporum* NRRL 26381/CL57 than in the pure culture.

Id	Product name/definition	Signal peptide	Transcriptome upregulation	Median pure culture	Median interaction	ECNum	Category	Fold Change
53742	Hypothetical protein	×	×					
73228	HpcH/HpaI aldolase/citrate lyase family protein	×	×					
44003	Hypothetical protein	√	√					
65799	Endoribonuclease L-PSP/chorismate mutase-like protein	×	×					
40665	Serine/threonine specific protein phosphatase	×	×			3.1.3.16	Hydrolase	
78957	ß-glucosidase	√	×			3.2.1.21	Hydrolase	
76038	ß-galactosidase	√	×			3.2.1.23	Hydrolase	
37457	Hypothetical protein	×	×					
41756	α/ß hydrolase fold protein-like protein	×	×					
78955	α/ß hydrolase fold	×	×					
87278	Formamidase	×	×			3.5.1.49	Hydrolase	
49010	Glutamate carboxypeptidase II (Peptidase M28)	×	×			3.4.17.21	Hydrolase	
50725	α-glucosidase	√	×			3.2.1.20	Hydrolase	
86981	Feruloyl esterase	√	×			3.1.1.73	Hydrolase	
84350	ß-galactosidase-domain containing protein	√	×					
68936	α/ß hydrolase fold	√	×					
61043	Hypothetical protein	√	√					
66348	Hypothetical protein	√	×					
67142	Putative ß-Ig-H3/fasciclin	×	×					
54208	Aminopeptidase I (Peptidase M18)	×	×			3.4.11.22	Hydrolase	
34126	α-mannosidase	×	×			3.2.1.24	Hydrolase	
79989	Pyridoxal phosphate-dependent transferase	×	×					
77891	Superoxide dismutase	×	×			1.15.1.1	Oxidoreductase	
76433	ß-glucosidase	√	×			3.2.1.21	Hydrolase	
79538	ß-ureidopropionase	×	×			3.5.1.6	Hydrolase	
54394	Hypothetical protein	×	×					
75855	Hypothetical protein	√	×					
79836	ß-lactamase/transpeptidase-like protein	×	×					
43783	Dihydroorotase	×	×			3.5.2.3	Hydrolase	
47165	Glycosyl hydrolase five-bladed β-propellor domain-containing	√	√					
59131	Gamma-glutamyltranspeptidase	×	×					
78756	Glycosyl hydrolase five-bladed β-propellor domain-containing	√	×					

0.005 0.6 1.5 11

Supplementary Table 9: Candidate hydrolase and secondary metabolite biosynthesis genes of *A. pullulans* NBB 7.2.1 involved in antagonism with *F.oxysporum* NRRL 26381/CL57.

**Hydrolases**				
Protein ID	Name	Transcriptome	Signal peptide	Proteome
67445	Arginase-family domain containing (Agmatinase)	✓	✓	
40837	Hypothetical proteinT ATP-ase	✓		
58691	Glycoside hydrolase\ α-L-arabinofuranosidase	✓		
48484	Mannan-endo-1,4-ß-mannosidase		✓	✓
67543	Arginase-family domain containing (Agmatinase)		✓	
78957	ß-glucosidase		✓	✓
76038	ß-galactosidase		✓	✓
50725	α-glucosidase		✓	✓
86981	Feruloyl esterase		✓	✓
76433	ß-glucosidase		✓	✓
Proteases				
49010	Peptidase M28			✓
54208	Aminopeptidase I (Peptidase M18)			✓
41106	Aspartic peptidase domain (Candidapepsin)	✓	✓	
22346	Aspartic peptidase domain (Yapsin 1)	✓	✓	
56152	Lysyl aminopeptidase	✓	✓	
56766	Secreted carboxypeptidase-like (Carboxypeptidase D)	✓	✓	
69063	Aspartic protease pepB (Aspergillopepsin I)	✓	✓	
**Secondary metabolite biosynthases**				
34549	hypothetical protein (NRPS)	✓		
78529	putative polyketide synthase	✓		
37339	Terpene synthase	✓		
39349	D-Lycopene ß-cyclase	✓		
49318	Peptide synthetase (NRPS)	✓		
